# The impact of price promotions on sales of unhealthy food and drink products in British retail stores

**DOI:** 10.1002/hec.4607

**Published:** 2022-10-02

**Authors:** Toby Watt, Walter Beckert, Richard Smith, Laura Cornelsen

**Affiliations:** ^1^ Department of Public Health Environments and Society Faculty of Public Health and Policy London School of Hygiene and Tropical Medicine (LSHTM) London UK; ^2^ The Health Foundation London UK; ^3^ Department of Economics Mathematics and Statistics Birkbeck University of London London UK; ^4^ College of Medicine and Health University of Exeter Exeter UK

**Keywords:** food policy, health economics, nutrition, obesity, price promotion

## Abstract

We study the health impact of food and beverage price promotion strategies—multi‐buy offers and price discounts, typically biased toward unhealthy product categories—in British consumer retail. We are the first to employ econometric models from the marketing literature to analyze the impact of price promotions with a focus on population health. Our dynamic, reduced form demand model incorporates endogenous inventory (stock piling), consumption rates imputed from repeat purchases and allows for unobserved household heterogeneity. We find that removing price discounts is more effective for reducing purchase volume compared to removing multi‐buy offers for 10 out of 12 food and drink groups, particularly those products for which price reduction is more common than multibuy. We find that price promotions induce consumption—and waste –through behavioral effects, associated with increased household inventory (stockpiling).

## 1 INTRODUCTION

In the UK 27% of adults are obese and a further 36% are overweight, which constitutes the highest level of obesity in Western Europe^1^. In England, nearly a quarter of children are obese or overweight by the age of five, and this rises to one third by age 11. In 2018 the Department of Health and Social Care (DHSC) set out a strategy to halve the rate of obesity among children by 2030^2^. As part of the strategy the DHSC announced in 2020 a ban of multi‐buy promotions for high‐in‐saturated‐fat, salt‐or‐sugar (HFSS) products which was set to take effect in 2022^3^. This policy was largely based on the work, published by Public Health England (PHE) (Smithson et al., 2015), showing that there are more promotions for unhealthy food than healthy food and that promotions can increase the amount of food purchased by around a fifth. However, this policy has now been called into question, at least in part as a reaction to the cost‐of‐living crisis (Smyth, 2022). This suggests *a fortiori* an evaluation is needed of the potential long‐term health costs of ill‐targeted promotions, to be able to balance them against short‐term pecuniary benefits.

**FIGURE 1:**
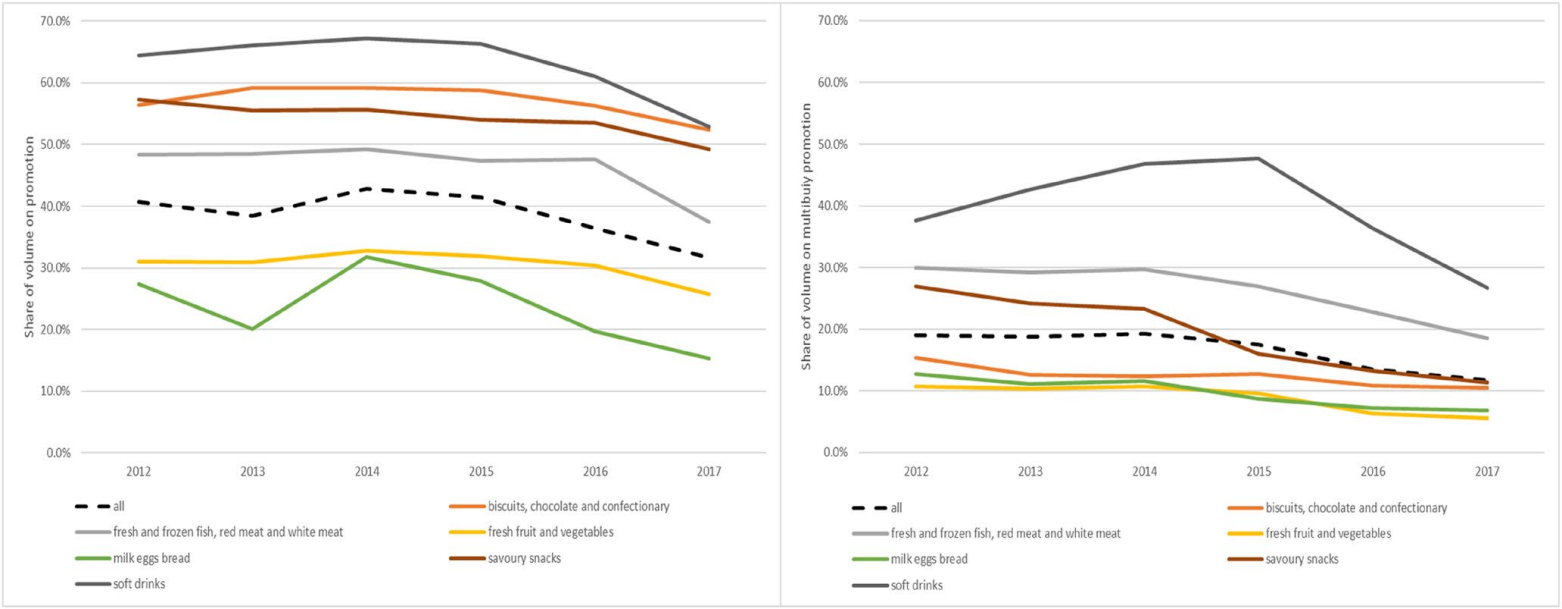
Share of volume of sales purchased on promotion by food/drink category (all promotions and multi‐buy promotions). Author's calculations from Kantar GB Fast Moving Consumer Goods (FMCG) panel

In Britain, price promotions are used as a key part of the marketing and competition strategy in national supermarket chains, as well as independent stores. Price promotions involve discounting prices at the point of sale and are available to all customers. They consist of total price reductions (TPRs), in which the good is offered at a price below recommended retail price, often presented as a percentage discount off the price or volume‐based discounts (multi‐buys), in which an additional percentage or whole units are offered upon purchase of a specified amount (e.g., buy‐one‐get‐one‐free deal, or 30% extra free). Both types of promotion are often indicated by brightly colored, large stickers which describe the terms of the price reduction. Analysis of Kantar GB Fast Moving Consumer Goods (FMCG) panel, a data set recording the shopping behavior of over 30,000 households in Britain, shows (Figure 1) that these promotions are common across food groups. Since 2014 there has been a reduction in the use of price promotions by about 10% points, with a concomitant decline in the share of goods bought through multi‐buy promotions but they remain high with at least a third of volume of food and beverages bought on some form of promotion^4^. There is clearly a large amount of variation across the food groups but longer lasting, less healthy categories such as soft drinks and snacks, compared with fresher, healthier products such as fruit and vegetables are consistently purchased more on promotion.

In light of the heavy use of promotions in British food retail and their policy relevancy, the aim of this paper is to estimate the extent to which price promotions—both total price reductions (TPRs) and multibuys—increase purchasing of potentially unhealthy food and drink items, including those that are frequently consumed by households with children (particularly children's cereal, children's biscuits and ketchup). We do this by estimating how price promotions influence a consumer's decision of when, what and how much to buy. Our reduced‐form demand model allows for an endogenous, dynamic consumption rate that is a function of the consumer's stockpiling decisions (the increased amount of goods held in the home beyond immediate needs). This allows us to infer possible overconsumption and the nutritional impact of price promotions.

Using the Kantar FMCG panel data on transaction level household food and beverage purchases from Britain between 2016 and 2017 to estimate our model, we find that, for all food and drink categories analyzed, purchases are made more frequently and a higher volume is purchased when goods are being price promoted. This effect occurs both for multi‐buy offers and TPRs, but TPRs typically exert a bigger effect on both. In agreement with literature we find that the consumption rate is not constant for a given household: the additional purchases are estimated to increase consumption of all food and drinks through the greater propensity to consume once the products are at home. Our results also suggest that the consumption rate of additional purchases depends on the type of product, with carbonated soft drinks, yoghurts, crisps, biscuits and children's cereal consumed more quickly once in the home than peanut butter, ketchup or canned baked beans.

Our analysis focusses on stockpiling of frequently purchased products and how inventory affects consumption behavior. As with other research attempting to model consumption dynamics, we look at households with high levels of consumption in the food and drink categories we analyze. From a public health perspective, it is also important to focus on these consumers: high consumers of sugary drinks and HFSS food, as they are at greater risk of developing poor health (Ebbeling et al., 2002; Pi‐Sunyer, 1991; Reilly et al., 2003).

We contribute to the literature in that we are the first to bring econometric methods, developed and used in marketing, to analyze a public health issue, focusing on the health implications for consumers rather than the sales implications for retailers. We also incorporate consumer level dynamics by estimating household inventory through repeat purchases at a household level, and we progress the methodology by adding household fixed effects to account for consumer level heterogeneity. Furthermore we are the first to present results of this kind of analysis on overall impact of consumption on food category rather than merely at brand level. We also estimate the impacts of multi‐buy promotions and TPRs separately.

We can conclude from our results that public health policy to restrict price promotions could be effective in reducing consumption of unhealthy food and drinks. Particularly it would be important to focus not only on multi‐buys but also on total price reductions as the latter appear more likely to cause over‐consumption or food waste.

## 2 LITERATURE

Hawkes (2009) highlighted that “*practically no attention has been paid to the impacts of sales promotions on dietary behaviors or how they could be used more effectively to promote healthy eating*”. Since then there has been growing public health policy interest (2018, Department of Health and Social Care, 2019, Chandon & Wansink, 2010). Some earlier theoretical work on the consumer response to price promotions (Chandon and Wansink, 2002, 2006) provided insight for other interventionist policies such as food vouchers (Griffith et al., 2018), restaurant food pricing and super‐sizing (Dobson & Gerstner, 2010) and nutritional labeling in supermarkets (Fichera and von Hinke, 2020). However, empirical research on the impact of price promotions has thus far looked at aggregate, market level analyses, grouping products into broad categories based on their nutritional value; looking at how price promotions are distributed between healthy and unhealthy products and finding that consumers are more responsive to price promotions on less healthy products (Nakamura et al., 2015; Smithson et al., 2015).

Given that most supermarket purchases are made for home consumption, the mechanism through which price promotions increase the level and rate consumption is through increasing household inventory. There are several theories to support this mechanism.

Consumers respond differently to price promotions depending on the good, its storability and their brand loyalty. Different products have different levels of sales volume on PP. Figure 1 shows that the share of purchases on promotion (by volume) across different food groups is typically higher for less healthy foods. Soft drinks and snacks are almost twice as likely to be bought on promotion in comparison to fruits and vegetables or, similarly storable but healthier, starchy foods.

However, a PP increasing purchases does not necessarily imply a one‐to‐one increase in consumption by the purchaser as it could simply be *advance* purchasing rather than *additional* purchasing. From a public health perspective, analyzing how this purchasing behavior translates to additional consumption behavior is the main interest. Research has sought to establish the impact of changing prices on food purchases, and research on price promotions typically refers to the increase in sales as a “promotion bump” (van Heerde et al., 2004). This within category sales bump can be attributed to three different forms of consumer reaction:

1. Consumer switching ‐ purchasing the same quantity but of a different brand. This has little nutritional effect;
2. Increased incidence or “impulse purchasing” ‐ promotions causing purchases that otherwise would not have occurred, creating an increase in consumption quantity;
3. Stockpiling ‐ increased purchase quantity in order to “take advantage” of a promotion and avoid higher spending on off‐promotion purchases in the future: meaning that purchases are greater than planned consumption in the short term. This *potentially* increases consumption of the category.

Consumer switching is essentially a cross‐price effect and does not affect the overall purchase quantity within a given category; therefore, these effects are less interesting from a nutritional health perspective. However, impulse purchasing and stockpiling fall into the category of “primary demand expansion”—an increase in the total volume of a product category purchased as a result of a promotion. The estimated size of the primary demand increase in recent studies is consistently larger than the consumer switching (Watt et al., 2020).

However, what does not necessarily follow from an expansion in purchasing from a price promotion is a corresponding increase in consumption^5^. The idea that price promotions change diets ‐ that promotions increase the consumption of a given food type, and do not simply change the timing of purchases or the goods selected ‐ hinges on present‐biased preferences, as well as stockpiling behavior. If having an abundance of a product available at home does not increase its consumption, then price promotions will simply bring purchases forward (intertemporal switching). In this case consumption remains constant over time and price promotions have no nutritional impact. However, if the rate of consumption is not constant, as the literature described in this section suggests, then price promotions could be leading to overconsumption and worse nutritional health; particularly if this occurs for unhealthy food. Thus, the relationship between stockpiling and increased consumption rate is key to understanding the impact of price promotions on population health.

Understanding the nutritional impact of price promotions also requires focus on the total sales of categories of foods or beverages, for example, the total purchases of cola flavored beverages, over a period of time. Whether brand A or brand B is selected is of second order importance. There are only a small number of researchers who have looked at overall category purchases (as opposed to focusing on individual brands). For example, Ailawadi and Neslin (1998) conduct simulations removing price promotions for randomly selected weeks, finding that, for yoghurt, 35% of the total promotion effect is caused by an increase in consumption (i.e., primary demand expansion). This is close to the 43% increase found by Sun (2005) for the same category who also found a 33% increase for canned tuna. Of the increased sales due to promotion in their study, Chan et al. (2008) estimate that 29% (18%) was an increase in consumption, for canned tuna (kitchen roll) (28% (14%) was brand switching and the remaining 43% (69%) was stockpiling). Ailawadi and Neslin (1998) found that ketchup consumption was relatively inflexible, with 12% of the promotion effect explained by increased consumption and Silva‐Risso et al. (1999) found, using the same model, that consumption of spaghetti sauce is similarly unresponsive.

Assunção and Meyer (1993) showed that, in markets where consumers are uncertain about future prices, consumption should rationally be higher if there is more household inventory and that, in the short‐term, households respond to promotions by buying more, but not necessarily eating and drinking more. In this they demonstrate that higher inventory levels allow a customer to consume at any desired rate, without having to return to the shops and possibly paying a higher replacement price. This is the reasoning drawn out in Ho et al. (1998)'s findings that stores with higher levels of price variation, for a given mean price, elicit higher levels of consumption from rational consumers. Folkes and Wheat (1995) posit a scarcity theory, similar to Wansink (1996) that within the household, smaller quantities are considered more valuable and therefore consumed more slowly. Chandon and Wansink (2002), by integrating key results from economic and mental accounting results, develop a salience‐convenience framework that indicates that exogenous product stockpiling can increase both frequency and quantity of eating and drinking at home by reducing the perceived acquisition and replacement costs of consumption. Stockpiled products can be more salient at the point of consumption because they take more storage space, can be unusually packaged (e.g., promotional packs), and can be placed in visible locations (on the counter vs. in the pantry) until depleted to a more typical level. Gourville and Soman (1998), Soman and John (2001) also propose that stockpiling can influence consumers’ perceptions of sunk costs of their inventory and therefore affects consumption rates (Gourville & Soman, 1998; Raghubir & Krishna, 1999; Soman & John, 2001; Wansink & Deshpande, 1994; Wansink et al., 2000).

## 3 DATA

Kantar GB FMCG data is a home‐scan consumer panel data set for food and beverages purchased in Great Britain (GB). The purchasing activity for “take‐home” food purchases are recorded by the panellists with hand‐held scanners, including information on expenditure on the product, whether the product was on promotion, type of promotion, volume bought, which shop it was bought from and the nutritional value of the product. The panel is a representative sample of British households comprising of about 30,000 households annually. Households are recruited via stratified sampling, with quotas set for region, household size, age of main shopper, number of children and occupation. Households record purchases continuously throughout the year. Panel retention is high—participating households in 2014 had median follow up time of 2.6 years (Berger et al., 2019). Approximately 3000–4000 new households are enrolled each year to maintain national representativeness. Panellists (main shopper in the household) provide socio‐demographic data when joining the panel (including age, sex, education occupational socio‐economic status, ethnicity, household size, presence of children, income bracket) followed by annual updates.

Using data from 2016 to 2017 we look at twelve different food and drink categories purchased from the five largest GB supermarket chains^6^. We analyze seven HFSS food products that are within the scope of Public Health England's Sugar and Calorie Reduction Program^7^ and two categories of sugar sweetened soft drinks: Children's cereal^8^, flavored yoghurt, crisps sold in multipacks for home consumption^9^, peanut butter, biscuits, cola flavored beverages and lemonade^10^. For comparison we also include three food categories that fall outside the scope, that are not HFSS products: baked beans, ketchup and unflavored (natural) yoghurt. These goods were also chosen because they are storable for more than 1 week either in a cupboard or the refrigerator and purchased by at least some consumers for frequent domestic consumption over a period of time.

Computationally, we are limited in the product groups that we can include. The greatest limitation lies in the number of potential alternatives (permutations of brand, flavor and size) within a food or drink category. For instance, we did not include chocolate confectionary because there are hundreds of different combinations of flavors and sizes of chocolate bars. To perform analysis on yoghurts we separate between natural and flavored (sweetened) yoghurts, and within flavored yoghurts we distinguish between multipacks or large (>400 g) individual pots^11^. We separate biscuits in to those which are marketed at children and everyday (sweet) biscuits^12^, using classification available within the data. In addition, we removed alternatives with the smallest sales by volume if the number of alternatives went beyond 80. These included everyday biscuits, crisps and each of the sub‐categories of yoghurt. To perform the analyses, the data are split into two time periods: 2016 is the initialization period, in which we identify, for each household, their loyalty to a given brand, loyalty to the format in which the product is sold, where appropriate (multipack, cans or bottles) and their average consumption rate. Then 2017 data are used to estimate the purchasing behavior model (see Methods). In our analysis we only include regular shoppers who have recorded at least one shopping trip to a supermarket per month for the 2‐year period, regardless of whether they bought any of the relevant goods on that shopping trip.

In order to understand the dynamics of households' intertemporal purchasing and consumption decisions it is important to focus on shoppers who frequently buy from the same product categories. From a public health perspective, it is also important to focus on these same consumers: for many, unhealthy products are a treat purchased sparingly. High consumers of sugary drinks and HFSS food, as we focus on here, are at greater risk of developing poor health. We therefore restrict the sample to the top tercile of households by purchased volume. To understand the robustness of our model, we perform the analysis on all consumers in our sample (with no lower limit to number of annual purchases), as well as the top tercile and top quintile of our households for sugar sweetened cola drinks. The ranking is based on number of packs purchased 2016–2017.

Volumes purchased, inventory and consumption are measured in centiliters (CL) or units (10 g) throughout. This is because the variable consumption function (Equation 3 in the Methods section) depends on the units used. Previous research in this area has been conducted in fluid ounces, roughly 3CL. Therefore, in order to verify and compare estimation results with the literature we have chosen to use a similar unit^13^. For each product type, Table 1 presents the number of consumers (households) in the sample, number of store visits and purchases, the total (by weight or volume) of the good purchased and what percentage of purchases by volume were made on promotion (TPR or multi‐buy). The minimum number of purchases per household for each group is presented in the last row. Of the products analyzed, only large individual pots of flavored yoghurt and everyday biscuits are purchased as frequently on multibuy promotion as TPR (Table 1). Baked beans, peanut butter and natural yoghurt had the lowest share of purchases made on (either type) promotion while five products from the HFSS categories, including lemonade, crisps and children's cereal had the highest shares of above 50% of purchases.

**TABLE 1:** Number of households, shop visit and purchase observations

	Sugar sweetened beverages				HFSS foods							Non‐HFSS foods		
	Cola (all)	Cola (top tercile)	Cola (top quintile)	Lemonade	Children's cereal	Flavored yoghurt (large pots)	Flavored yoghurt (multi pack)	Crisps (multi pack)	Children's biscuits	Everyday biscuits	Peanut butter	Baked beans	Ketchup	Natural yoghurt
# Households	1638	811	501	1021	1720	1039	697	899	1471	1509	972	2722	1791	1882
% Households with children	33%	35%	35%	35%	47%	29%	23%	17%	63%	24%	35%	31%	41%	28%
# Store visits	133,467	68,313	42,948	87,692	139,783	82,545	53,237	71,716	119,839	118,904	80,136	224,196	146,889	152,227
# Purchases	11,152	9568	8123	8875	15,031	10,133	9446	11,830	15,194	17,924	6578	34,450	9575	28,770
Mean annual spend per hh (£)^a^	20.27	35.00	48.14	10.22	23.34	19.04	30.00	24.27	14.57	12.17	13.00	14.22	10.26	24.19
Mean annual volume per hh (L/Kg)^b^	28.14	49.53	69.49	25.66	6.46	7.75	14.04	3.59	2.30	6.85	6.56	14.01	3.91	10.64
% Purchase (count): Multibuy^c^	7.4%	8.0%	8.4%	6.9%	6.7%	36.2%	6.0%	20.4%	0.9%	15.0%	0.2%	4.5%	0.2%	2.6%
% Purchases (count): Total price reduction^d^	40.8%	39.2%	38.6%	53.0%	54.2%	32.8%	42.7%	40.8%	65.6%	15.3%	19.1%	31.0%	57.3%	19.6%
Total bought on promotion	48.2%	47.2%	47.0%	59.9%	60.9%	69.0%	48.7%	61.3%	66.6%	30.3%	19.3%	35.4%	57.5%	22.2%
Min # of purchases per year (average, 16–17)	1	4.5	8.5	4	5.5	10.5	5.5	6.5	6	7	3.5	10	3	6.5

^a^ Annual spend and volume per household for each food category.

^b^ Annual spend and volume per household for each food category.

^c^ Multi‐buys are volume based discounts, in which an additional % or additional units are offered upon purchase of a specified amount (e.g., buy‐one‐get‐one‐free deal, or 30% extra free).

^d^ Total price reductions (TPR), in which the good is offered at a price below recommended retail price, often presented as a % discount off the price.

Summaries of the used variables and the incidence and quantity decision outcomes for the initialization period are presented in Annex 1.

Due to the nature of the data we only obtain unit prices, calculated from expenditure and volume information for each purchase, and we observe in the data the existence of promotions when a purchase is made, hence the precise set of offers and prices are unknown from the data alone. However, we are able to overcome this issue due to a feature of the way GB supermarkets operate, where major supermarkets operate a country‐wide pricing and promotion strategy (Commission, 1999; Competition and Markets Authority, 2008; Thomassen et al., 2017). This means in practice that same types of stores in the same chain have constant prices (i.e., price does not vary by geography). Utilizing this feature, we impute the prices of non‐purchased items in the category at a given time through the purchases of other shoppers in the data set. Due to a lack of stock information we are, however, required to assume that goods are always available when consumers visit the store. We believe this to be a reasonable assumption for our categories given their popularity. Coverage using this method is not perfect and therefore some prices must be imputed at a supermarket chain/type‐of‐store level; for example, if an item had been bought sometime by some consumer before and by some consumer after at the same price at a given store then we assume that the price is consistent in‐between purchases. If the price is different then we swap the prices over mid‐way between the observed prices.

## 4 METHODS

In order to analyze price promotions by product at a household level, we make use of empirical methods from the marketing literature that exploit panel sales data. This extensive literature began with differing models from Gupta (1988) and Chintagunta (1993). Gupta developed a reduced form framework that estimates the underlying purchasing decisions of when, what and how much to buy ‘separately’, while Chintagunta modeled all three under a ‘single utility’ model. This literature is extensively reviewed in Hawkes (2009), Neslin and van Heerde (2009) and (Watt et al., 2020).

Our analysis focuses on the measurement of the short‐term effects of price promotions, as does much of the literature, primarily because for many products the market is characterized by frequent short‐term promotions. The theoretical basis for this is demonstrated by Ho et al. (1998) who ﬁnd that consumption should increase for products with higher levels of price variation, for a given mean price across stores for a frequently purchased product.

Many models of price promotions attempt to account for the dynamic issues raised in the early literature. Those models that incorporate dynamics in a unified utility model are necessarily complex dynamic structural models. These models initially required, for tractability, that consumption be exogenous and constant (Erdem et al., 2003; Hendel & Nevo, 2006). Sun (2005) developed a dynamic structural model with endogenous consumption, with similar work to follow by Chan et al. (2008). These models were considered and remain ‘*state of the art in analyzing purchase behavior in a single product category*’ (Song & Chintagunta, 2007). They show that static demand models will overestimate cross‐price elasticities if consumers are engaged in stockpiling behavior and will overestimate the long‐run sales increase associated with price promotions. In an attempt to account for these dynamics, but in more tractable reduced form models Ailawadi and Neslin (1998), Bell and Boztuğ (2007), Ailawadi et al. (2007) and Neslin and van Heerde (2009) have introduced dynamics through imputation of household inventory and the presence of brand loyalty in the brand decision estimation.

We use the reduced form model presented in Ailawadi and Neslin (1998) (henceforth AN) and others (Ailawadi et al., 2007; Ailawadi & Neslin, 1998; Bell & Boztuğ, 2007; Neslin and van Heerde, 2009). We account for unobserved consumer heterogeneity by introducing consumer level fixed effects, which greatly improves model fit as presented in our results section.

At any given shop visit *t*, a PP can influence a customer's decision to purchase, the brand they choose and the quantity they buy. The probability that consumer *h* buys *q* units of good *b* on their visit to a store *t* can be expressed:

(1) $$P\left(Q_{bt}^{h}=q_{bt}^{h}\right)=P\left(I_{t}^{h}=1\right)*P\left(C_{t}^{h}=b|I_{t}^{h}=1\right)*P\left(Q_{bt}^{h}=q_{t}^{h}|I_{t}^{h}=1,B_{t}^{h}=b\;\right)$$

where:

$P\left(I_{t}^{h}=1\right)$ is the probability that the binary random variable *I*_*t*_^*h*^ = 1, indicating that consumer *h* makes a purchase of the product category on visit *t*

$P\left(C_{t}^{h}=b|I_{t}^{h}=1\right)$ is the probability that consumer *h* chooses brand *b* conditional on their decision to make a purchase

$P\left(Q_{bt}^{h}=q_{bt}^{h}|I_{t}^{h}=1,C_{t}^{h}=b\;\right)$ is the probability that consumer *h* buys $q_{bt}^{h}$ units of the good, conditional on their brand choice and making a purchase^14^.

We model purchase incidence, brand choice and quantity purchased separately for a given shopping trip. An important part of both the incidence and quantity decision is each consumer's rate of consumption and the inventory they hold at any given time. In the fashion of AN we discuss the process for identifying inventory and flexible consumption rate.

### 4.1 Inventory

We follow the methods of many researchers in the marketing literature by recursively calculating inventory levels for each consumer over time based on purchases made and an assumed rate of consumption (Ailawadi et al., 2007; Ailawadi & Neslin, 1998; Bell & Boztuğ, 2007; Bucklin & Lattin, 1991; Chan et al., 2008; Chintagunta, 1993; Gupta, 1988; Neslin & van Heerde, 2009; Sun, 2005). The evolution of inventory is modeled as:

(2) $${inv}_{d}^{h}\equiv {inv}_{d-1}^{h}+{PurQty}_{d-1}^{h}-{consumption}_{d-1}^{h}$$

where:

${inv}_{d}^{h}$ is the inventory of consumer *h* at the beginning of day *d*.

${PurQty}_{d-1}^{h}$ is the quantity of the category purchased by consumer *h* at on day *d*−1^15^,

${consumption}_{d-1}^{h}$ is the quantity of the category consumed by consumer *h* at on day *d*−1.

We use *d* to denote the day, as opposed to *t* in other equations, as inventory and consumption are estimated daily, while purchasing decisions only occur when a store is visited. We assume that each consumer enters the observation horizon with 2 weeks' worth of their average consumption as their inventory, however the value of the starting inventory does not quantitatively affect our results, as is consistent with the literature (Ailawadi & Neslin, 1998; Bell & Boztuğ, 2007; Erdem et al., 2003). Therefore, the starting inventory is 14 times the average daily consumption rate (${inv}_{1}^{h}={\bar{cons}}^{h}*14$), where ${\bar{cons}}^{h}$ is the average daily consumption rate, calculated as the total volume purchased divided by the number of days within the initialization period. Importantly this assumption means we do not allow for waste in any explicit way. This is an insurmountable shortcoming of the data; however, we would suggest that the waste of food and drink is equally a negative outcome, which should also be considered in the context of price promotions and public policy.

### 4.2 Consumption rate

The AN assumption is that consumption follows a continuous non‐linear function that increases in inventory on a given day and the average level of consumption, of the form:

(3) $${cons}_{d}^{h}={inv}_{d}^{h}\left[\frac{{\bar{cons}}^{h}}{{\bar{cons}}^{h}+{\left({inv}_{d}^{h}\right)}^{\psi }}\right]$$

Where ψ is a single parameter i.e., restricted to be less than 1 and can be negative. It governs the extent to which consumption increases with higher levels of inventory.

AN justify the use of this function because it has the following desirable characteristics.

(i) It is parsimonious with only a single parameter to be estimated;
(ii) Consumption does not exceed inventory, removing the need to truncate the function;
(iii) High values of ψ imply less flexible consumption because consumption initially increases with inventory and then levels off. Low values of ψ imply flexible consumption, where consumption continually increases with inventory.
(iv) For a given value of ψ, consumers with a high ${\bar{cons}}^{h}$ consume more than low users for any given level of inventory, as displayed in Annex 2;

For a given value of average consumption, the value of ψ determines the extent to which consumption responds to inventory. A value of ψ close to one indicates that consumption is close to constant at the average rate of consumption. A lower value of ψ increases the extent to which consumption depends on inventory.

We use a different probability model for estimation of each of the three decisions. The purchase incidence follows a binary logit, the brand choice a McFadden choice model (Mcfadden, 1973), which is a form of conditional logit model which allows for case specific fixed effects and a truncated Poisson distribution for the quantity decision. In the brand choice decision, we calculate the expected value of making a purchase for every shop visit: this expected value from the brand choice decision affects the likelihood of purchase incidence through the “inclusive value” (Ailawadi & Neslin, 1998; Hausman et al., 1994; Hendel & Nevo, 2006). Reduced‐form modeling of consumer decisions creates several issues of econometric identification. In terms of prices and price promotions our model requires three assumption:

A1. Price promotion setting is exogenous.

Price exogeneity, the notion that once we condition on observed variables and household fixed effects the price process is independent of the unobserved random elements of the model, is pervasively assumed in the literature (Ailawadi & Neslin, 1998; Bell & Boztuğ, 2007; Chan et al., 2008; Ebling & Klapper, 2010; Erdem et al., 2003; Hendel & Nevo, 2006). We use their same justifications: we are using household‐level data and we control for unobserved household characteristics, so if promotions are targeted to certain factions of the market that dependence of promotions will be absorbed. The main concern is seasonality of purchase decisions, with promotions targeted at periods of high consumption when the likelihood of a sale changes: we model seasonality using quarterly dummies in our purchase incidence decision to control for this.

A2. Observed price changes are attributable to price promotions not to changes in supply.

**FIGURE 2:**
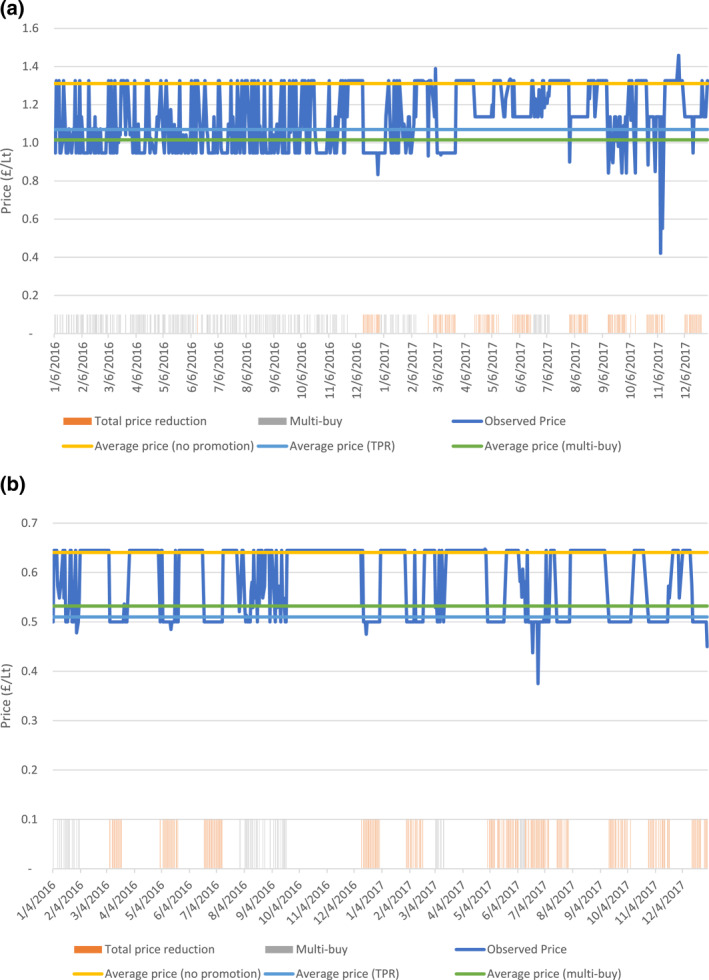
(a) Prices over time for an 8 pack of 330 ml cans of cola, with average prices per promotion by type. The blue line denotes the observed prices, TPR (Total price reductions) and Multi‐buy promotions are signified using colors bars at the bottom, the average price for promoted and non‐promoted are denoted by horizontal lines. (b) Prices over time for 2Lt bottle of a single brand of lemonade, with average prices per promotion by type. The blue line denotes the observed prices, TPR (Total price reductions) and Multi‐buy promotions are signified using colors bars at the bottom, the average price for promoted and non‐promoted are denoted by horizontal lines

In the data we observe frequent price promotions for individual goods. For the goods analyzed here over our 2 years of data the price variation observed is either completely or almost completely driven by price promotions as observed in Figure 2 below. As an example, we show the weekly price of a particular brand of cola drink served in 2lt bottles at a single British supermarket. We see that the non‐promoted price per liter is almost constant, with the price variation driven wholly by promotions.

The third identification issue associated with the reduced form demand model is the separation of the three purchasing decisions. Particularly there are identification issues associated with the use of the same price changes in multiple models concurrently that is,

A3. Having decided to make a purchase, the impact of price promotions on decision of how much to buy is independent of the impact of price promotions on the decision to make a purchase.

An important analysis of the relative merits of the ‘separate’ (reduced form) and the ‘single utility’ (structural) models has been performed by Ebling and Klapper (2010). They find that, for the beverage market, unifying does improve model fit over the separable model. However, they conclude that all models present results that are ‘*quite intuitive and in line with the characteristics of the data… and that using one model over another causes no substantial harm*’. We therefore feel confident that this model is appropriate to analyze the impact of price promotions.

The remainder of this section outlines the specification of the models for purchase incidence, brand choice and quantity decisions.

### 4.3 Purchase incidence model

Product purchase incidence is modeled as a binary logit with household level fixed effects.

(4) $$P\left(I_{t}^{h}=1\right)=\frac{1}{1+e^{-{Utility\;of\;incidence}_{t}^{h}}}\;$$

where Utility of incidence is modeled in the following way:

(5) $${Utility\;of\;incidence}_{t}^{h}=V_{t}^{h}=\gamma_{1}{inv}_{t}^{h}+\gamma_{2}{incl\_val}_{t}^{h}+\gamma_{3}{inc}_{t-1}^{h}+\sum_{i=1}^{3}\gamma_{4i}Q_{t}^{h}+\gamma_{0}^{h}+\varphi_{t}^{h}$$

where, ${\mathrm{inv}}_{t}^{h}$ is their inventory just before the visit *t* (as described in Equation 3), ${inc}_{t-1}^{h}$ is whether the consumer purchased any of the category on their last shopping trip and *incl*_*val*_*t*_^*h*^ is the inclusive value, or category value of the brand selection model (Equation 6) for consumer *h* on visit *t* defined as:

(6) $${incl\_val}_{t}^{h}=\ln\left(\sum_{b=1}^{B}e^{{Utility\;of\;brand\;choice}_{bt}^{h}}\right)$$

where ${Utility\;of\;brand\;choice}_{bt}^{h}$ is defined in the next section. Within the multinomial logit framework, this inclusive value is thought of as the expected utility from making a purchase, based on the prices, promotions and products that the shopper is presented with on their trip (B, *the product range available in the store*). We can think of it as the expected value of making a purchase.

We include quarterly dummies $Q_{t}^{h}$, a fixed effect for each consumer and a mean zero, type I extreme value, exogenous and homoscedastic error term, *φ*_*t*_^*h*^. We use stata command xtlogit, fe to estimate the purchase decision.

By including the inclusive value from the brand choice in our incidence model we are able to allow for prices and promotions to influence the purchase decision indirectly.

### 4.4 Brand choice model

The brand choice decision is taken conditional on a purchase being made. Therefore, there is no outside option and we follow a conditional logit model. The probability of consumer *h* selecting brand *b* at time *t*, is conditional on purchase incidence as follows:

(7) $$P\left(C_{t}^{h}=b|I_{t}^{h}=1\right)=\frac{e^{{Utility\;of\;brand\;choice}_{bt}^{h}}}{\sum_{b^{\prime }=1}^{B}e^{{Utility\;of\;brand\;choice}_{b^{\prime }t}^{h}}}$$

where:

(8) $${Utility\;of\;brand\;choice}_{bt}^{h}=U_{bt}^{h}=\alpha_{0b}+{\alpha_{1}\;\ln(Price}_{bt}^{h})+\alpha_{2}{TPR}_{bt}^{h}+\alpha_{3}{MultiBuy}_{bt}^{h}+{\alpha_{4}Last}_{bt}^{h}+{\alpha_{5}BrandLoyalty}_{b}^{h}+{\alpha_{6}TypeLoyalty}_{b}^{h}+{\alpha_{7}PackLoyalty}_{b}^{h}+{\alpha_{8}FlavourLoyalty}_{b}^{h}+\alpha_{9b}{\bar{cons}}^{h}+\alpha_{t}^{h}+\varepsilon_{t}^{h}$$

where *α*_0*b*_ is a brand specific constant term, ${price}_{bt}^{h}$ is the price of brand in the supermarket that consumer *h* visited at time *t*, promotion dummy variables are split into two types, price reduction (TPR) and multi‐buy, ${\alpha_{3}Last}_{bt}^{h}$ is a dummy stating whether the brand was purchased by the consumer on their last shopping trip, ${BrandLoyalty}_{b}^{h}$, ${TypeLoyalty}_{b}^{h}$, ${PackLoyalty}_{b}^{h}$ and ${FlavourLoyalty}_{b}^{h}$ are time invariant consumer specific variable indicating the portion by volume of brand, type^16^, the packaging^17^ and flavor^18^ of *b* as a total purchases of the consumer over the initialization period.

The constant term and the coefficient on average daily consumption from the initialization period $\left({\bar{cons}}^{h}\right)$ are allowed to vary by brand *b*. *α*_*t*_^*h*^ is the consumer and shop visit, consumer‐shop fixed effect, and *ε*_*t*_^*h*^ are independent Type I (Gumbel‐type) extreme‐value random variables with mean γ (the Euler–Mascheroni constant, approximately 0.577) and variance π 2/6. Stata's command asclogit is used for the estimation (.Statacorp).

### 4.5 Purchase quantity model

Conditional on the brand‐type‐size combination chosen by household *h*, and the fact that they have chosen to make a purchase of the product, the consumer must choose the number of units (bottles, cans, packs) they wish to buy. Note that the quantity here is in units of brand/size. For example, when product is cola (cereal) in a 2LT bottle (500 g box) a *q* = 2 means 2 × 2LT bottle (2 × 500 g box) has been purchased. This in turn implies a total volume of 4LT (1 kg) or 400CL (100 units of 10 g).

A truncated‐at‐zero Poisson model governs probability of purchasing *q* units (*q* > 0), given that the purchase incidence and brand decision hurdle has been passed:

(9) $$P\left(q_{t}^{h}\left|I_{t}^{h}=1\right.\right)=\frac{{\left(\lambda_{t}^{h}\right)}^{q_{t}^{h}}}{\left(e^{\lambda_{t}^{h}}-1\right)q_{t}^{h}!}$$

where the Poisson parameter $\lambda_{t}^{h}$ is a linear function of (mean‐centered) inventory, the size, price, and promotion status of the selected brand‐size. Due to computational limitations we were not able to estimate this truncated Poisson using fixed effects. Instead we perform a least squared dummy variables type truncated Poisson estimation using Stata's tpoisson command, incorporating the Mundlak method (Mundlak, 1978). The Mundlak method includes the means of the explanatory variables, clustered by household. This approximates the time invariant individual effects as a function of individual means of time varying characteristics. We also cluster standard errors by household.

(10) $$\lambda_{t}^{h}=\phi_{1}{\ln(Price}_{t}^{h})+\phi_{2}{TPR}_{t}^{h}+\phi_{2}{MultiBuy}_{t}^{h}+\phi_{4}{INV}_{t}^{h}+\phi_{5}{PackSize}_{t}^{h}+\phi_{6}{\bar{\ln\left(Price\right)}}^{h}+\phi_{7}{\bar{TPR}}^{h}+\phi_{8}{\bar{MultiBuy}}^{h}+\phi_{9}{\bar{INV}}^{h}+\phi_{10}{\bar{PackSize}}^{h}+\phi_{11}{\bar{Cons}}^{h}+\epsilon_{t}^{h}$$

Where, $\ln\left({Price}_{t}^{h}\right)$ is the natural log of the price paid by the consumer at time *t*; ${\text{TPR}}_{t}^{h}$ is a dummy variable equal to 1 where the good purchased is on price reduction, 0 otherwise; ${\text{MultiBuy}}_{t}^{h}$ is a dummy variable equal to 1 where the good purchased is on multi‐buy promotion, 0 otherwise; *INV*_*t*_^*h*^ is the same inventory level as presented in the incidence model; PackSize_*t*_^*h*^ denotes the volume of the chosen package; ${\bar{\ln\left(Price\right)}}^{h},\;{\bar{TPR}}^{h},\;{\bar{MultiBuy}}^{h},$ ${\bar{INV}}^{h},\;{\bar{PackSize}}^{h}$are the averages of the corresponding explanatory variable by consumer; ${\bar{Cons}}^{h}$ is the average level of consumption from each consumer from the 2016 data; *ϵ*_*t*_^*h*^, is a normally distributed error term that is mean zero and allowed to be correlated for within consumer observations.

We estimate the three decisions above using a likelihood maximization set out in AN (see Annex 3 for estimation details). For comparison to flexible consumption rate model we also estimate one with constant consumption rate at the household's average rate of consumption in the initialization period.

Finally, in our calculation of the overall impact of price promotion on sales volumes (Table 4) we calculate semi‐elasticities for the incidence model (Equation (4), using the stata command aextlogit (Kemp & Santos Silva, 2016; Santos Silva, 2016)) and marginal effects for the impact of price changes on quantity purchased model (Equation (9), using the stata command margins with zero‐truncated conditional means).

## 5 RESULTS

### 5.1 Model fit

Table 2 (split into a and b) shows the relative likelihoods from estimation processes of flexible and constant consumption rate, respectively, for the food and beverage groups under study. As with other research in using these methods (Ailawadi et al., 2007; Ailawadi & Neslin, 1998; Chan et al., 2008; Sun, 2005), we find that a flexible rate of consumption underlying the estimation of an inventory significantly improves the overall fit of the model when compared to a model of constant consumption rate. The likelihood ratio (L‐R) tests confirms that there is an improvement for all products analyzed.

**TABLE 2:** (a and b) Comparison of goodness of fit for flexible consumption

	Cola (all)		Cola (top tercile)		Cola (top quintile)		Lemonade		Children's cereal		Flavored yoghurt (large pots)		Flavored yoghurt (multi pack)	
	Const cons (2017)	Flex cons	Const cons (2017)	Flex cons	Const cons (2017)	Flex cons	Const cons (2017)	Flex cons	Const cons (2017)	Flex cons	Const cons (2017)	Flex cons	Const cons (2017)	Flex cons
Purchase incidence estimation														
# Households	1638		811		501		1021		1720		1039		697	
# Observations	133,467		68,313		42,948		87,692		139,783		82,545		53,237	
Log‐likelihood	−26,405	−26,318	−20,391	−20,305	−15,765	−15,684	−21,710	−21,675	−38,590	−38,555	−23,102	−22,996	−18,229	−18,114
Quantity purchased estimation														
# Observations	11,152		9568		8123		8875		15,031		10,133		9446	
Log‐likelihood	−8075	−7799	−7423	−7189	−6726	−6521	−6843	−6867	−8040	−7986	−9464	−9453	−7411	−7472
Overall model														
Parameter ‐ *f* value	‐	0.13	‐	0.12	‐	0.16	‐	−0.10	‐	−0.10	‐	−2.62	‐	−0.17
Log‐likelihood	−34,480	−34,117	−27,814	−27,495	−22,491	−22,204	−28,554	−28,541	−46,630	−46,541	−32,566	−32,449	−25,640	−25,586
L‐R Test stat	725.71		638.51		572.98		24.24		179.63		234.13		108.91	
*p*‐value	<0.001		<0.001		<0.001		<0.001		<0.001		<0.001		<0.001	

	Crisps (multi pack)		Children's biscuits		Everyday biscuits		Peanut butter		Baked beans		Ketchup		Natural yoghurt	
	Const cons (2017)	Flex cons	Const cons (2017)	Flex cons	Const cons (2017)	Flex cons	Const cons (2017)	Flex cons	Const cons (2017)	Flex cons	Const cons (2017)	Flex cons	Const cons (2017)	Flex cons
Purchase incidence estimation														
# Households	899		1471		1509		972		2722		1791		1882	
# Observations	71,716		119,839		118,904		80,136		224,196		146,889		152,227	
Log‐likelihood	−24,877	−24,816	−35,877	−35,869	−39,565	−39,482	−18,075	−18,069	−76,111	−75,964	−28,021	−28,014	−57,342	−57,061
Quantity purchased estimation														
# Observations	11,830		15,194		17,924		6578		34,450		9575		28,770	
Log‐likelihood	−9487	−9416	−14,064	−14,001	−17,378	−17,322	−2152	−2142	−29,308	−29,343	−1633	−1631	−19,007	−18,691
Overall model														
Parameter‐*f* value	‐	−0.61	‐	−3.09	‐	−0.32	‐	0.93	‐	0.60	‐	0.82	‐	−0.45
Log‐likelihood	−34,364	−34,231	−49,942	−49,870	−56,944	−56,805	−20,227	−20,212	−105,418	−105,307	−29,655	−29,645	−76,349	−75,752
L‐R Test stat	265.72		143.37		277.47		30.81		223.73		19.10		1194.25	
*p*‐value	<0.001		<0.001		<0.001		<0.001		<0.001		<0.001		<0.001	

*Note*: Model fit results from the reduced form model of each food and drink category, showing an improvement of fit associated with flexible consumption rates. The L‐R Test Stat (Likelihood ratio test statistic) follows a Chi‐Squared distribution with two degrees of freedom: the inventory model is affected by the flexible consumption model and enters our model of purchasing twice.

We further perform a likelihood ratio test on the combined likelihoods of the incidence and quantity model to compare the fit of the models under constant and flexible consumption (brand choice is assumed not to be affected by inventory). The L‐R test rejects H0 that consumption (eating, drinking or waste) is constant and does not depend on inventory for all products. Ketchup and peanut butter are estimated to be the most inflexible (ψ closest to 1) which aligns with the dynamic demand estimation literature that frequently use ketchup (or toilet roll or laundry detergent) as an example of a product for which little or no additional utility is gained from consuming more.

The majority of the fit improvement (increase in likelihood) is contained in the purchase incidence portion of the model. The improved fit of the model in allowing for flexible consumption is driven by the inventory coefficient in the incidence regression. This implies that remaining inventory is more important in the decision of whether to make a purchase and less important when consumers decide how much to purchase.

We can also observe the importance of consumer heterogeneity when we compare groups of cola shoppers: households that on average purchase greater volumes are estimated to be less flexible in their consumption (estimates of the parameter ψ are closer to 1). This implies that their estimated consumption rate depends less on their inventory: consumers with consistently higher levels of household inventory are able to maintain a consistent (high) level of consumption without fear of stock out.

Finally, in Table 3 we show the extent of the improvement of model fit across all products for the flexible consumption by including fixed effects.

**TABLE 3:** Comparison of goodness of fit for fixed effects

	Sugar sweetened beverages				HFSS foods							Non‐HFSS foods		
	Cola (all)	Cola (top tercile)	Cola (top quintile)	Lemonade	Children's cereal	Flavored yoghurt (large pots)	Flavored yoghurt (multi pack)	Crisps (multi pack)	Children's biscuits	Everyday biscuits	Peanut butter	Baked beans	Ketchup	Natural yoghurt
Log‐likelihood with flexible consumption	−34,117	−27,495	−22,204	−28,541	−46,541	−32,449	−25,586	−34,231	−49,870	−56,805	−20,212	−105,307	−29,645	−75,752
Log‐likelihood without FE, with flexible consumption	−44,189	−32,558	−25,862	−33,684	−54,219	−37,889	−29,663	−39,259	−57,534	−64,598	−24,668	−121,567	−35,751	−88,108
L‐R Test stat	20,145	10,126	7315	10,286	15,357	10,879	8153	10,056	15,328	15,586	8913	32,522	12,212	24,712
*p* value	<0.001	<0.001	<0.001	<0.001	<0.001	<0.001	<0.001	<0.001	<0.001	<0.001	<0.001	<0.001	<0.001	<0.001

*Note*: Model fit results from the reduced form model of each food and drink category, showing an improvement of fit associated with allowing for household heterogeneity through fixed effects. The L‐R Test Stat (Likelihood ratio test statistic) follows a Chi‐Squared distribution with two degrees of freedom: the inventory model is affected by the flexible consumption model and enters our model of purchasing twice.

### 5.2 Impact of price promotions

We briefly discuss the direction and significance of the coefficients in each of the three decision models (as the magnitude of the coefficients is not directly interpretable) and then focus on the analysis of elasticities and marginal effects of promotions and associated price changes on purchase volumes. Detailed tables of the regression models from each of the three decisions are shown in Annex 4 and volume effect results are presented in Table 4.

**TABLE 4:** Impact of promotions on volume sales

			Sugar sweetened beverages				HSSF foods							Non‐HSSF foods		
			Cola (all)	Cola (top tercile)	Cola (top quintile)	Lemonade	Children's cereal	Flavored yoghurt (large pots)	Flavored yoghurt (multi pack)	Crisps (multi pack)	Children's biscuits	Everyday biscuits	Peanut butter	Baked beans	Ketchup	Natural yoghurt
Number of consumers			1638	811	501	1021	1720	1039	697	899	1471	1509	972	2722	1791	1882
Number of store visits			133,467	68,313	42,948	87,692	139,783	82,545	53,237	71,716	119,839	118,904	80,136	224,196	146,889	152,227
Number of purchases			11,152	9568	8123	8875	15,031	10,133	9446	11,830	15,194	17,924	6578	34,450	9575	28,770
Volume purchased			14,242	13,330	12,298	12,838	18,748	16,541	13,581	18,361	23,965	31,484	7532	60,289	10,181	37,510
% Purchase incidence			8.4%	14.0%	18.9%	10.1%	10.8%	12.3%	17.7%	16.5%	12.7%	15.1%	8.2%	15.4%	6.5%	18.9%
Share of incidence bought on promotion	TPR		40.8%	39.2%	38.6%	53.0%	54.2%	32.8%	42.7%	40.8%	65.6%	15.3%	19.1%	31.0%	57.3%	19.6%
	Multibuy		7.4%	8.0%	8.4%	6.9%	6.7%	36.2%	6.0%	20.4%	0.9%	15.0%	0.2%	4.5%	0.2%	2.6%
Average purchase quantity per incidence			1.28	1.39	1.51	1.45	1.25	1.63	1.44	1.55	1.58	1.76	1.15	1.75	1.06	1.30
Mean inclusive value from brand choice			17.64	17.73	18.62	13.19	8.39	9.64	10.09	10.97	12.89	8.32	9.81	17.14	14.30	10.35
			(0.75)	(0.8)	(0.85)	(1.03)	(0.96)	(0.87)	(1.25)	(0.82)	(1.25)	(0.7)	(1.43)	(1.21)	(1.07)	(2.08)
Mean inclusive without promotion	TPR		17.27	16.76	17.60	11.67	7.60	9.12	9.21	10.12	11.72	8.09	9.54	16.50	12.99	10.05
			(0.73)	(1.14)	(1.25)	(1.54)	(0.95)	(0.99)	(1.44)	(1.12)	(1.24)	(0.74)	(1.56)	(1.36)	(1.15)	(2.09)
	Multibuy		17.60	17.56	18.41	12.96	8.20	8.97	10.00	10.44	12.87	8.00	9.81	17.06	14.30	10.31
			(0.75)	(0.92)	(0.999)	(1.14)	(0.93)	(0.93)	(1.29)	(1.03)	(1.25)	(0.85)	(1.43)	(1.26)	(1.08)	(2.09)
Purchase incidence	Semi‐elasticity of inclusive value (at means) on purchase incidence		0.149	0.240	0.255	0.224	0.115	0.355	0.410	0.190	0.269	0.175	0.136	0.332	0.347	0.136
			(0.0215)	(0.0211)	(0.0214)	(0.0206)	(0.0113)	(0.0213)	(0.0228)	(0.0193)	(0.0219)	(0.0187)	(0.0223)	(0.0089)	(0.0173)	(0.0121)
	Percentage reduction in incidence from removing	TPR	**5.5%**	**23.3%**	**26.0%**	**33.9%**	**9.0%**	**18.4%**	**36.2%**	**16.1%**	**31.3%**	**4.0%**	**3.7%**	**21.5%**	**45.8%**	**4.0%**
		Multibuy	**0.7%**	**4.2%**	**5.4%**	**5.0%**	**2.2%**	**23.6%**	**3.9%**	**9.9%**	**0.4%**	**5.6%**	**0.02%**	**2.9%**	**0.3%**	**0.5%**
Purchase quantity	Coefficient estimate for quantity choice for promotion dummy	TPR	0.091	0.105	0.115	0.079	0.522	0.299	0.337	0.361	0.277	0.316	0.672	0.269	0.736	0.230
			(0.05)	(0.05)	(0.05)	(0.04)	(0.07)	(0.06)	(0.06)	(0.07)	(0.03)	(0.04)	(0.13)	(0.02)	(0.14)	(0.04)
		Multibuy	0.330	0.317	0.291	0.489	1.393	0.914	0.822	1.064	0.844	0.675	2.675	0.745	2.534	0.815
			(0.07)	(0.07)	(0.06)	(0.08)	(0.1)	(0.08)	(0.08)	(0.08)	(0.09)	(0.04)	(0.21)	(0.04)	(0.51)	(0.09)
	Marginal effect for quantity choice ln(Price)		−0.107	^a^	^a^	−0.131	−0.157	−0.256	−0.144	−0.146	−0.133	−0.275	−0.124	−0.250	^a^	−0.072
			(0.06)	(0)	(0)	(0.05)	(0.03)	(0.1)	(0.05)	(0.07)	(0.05)	(0.04)	(0.03)	(0.04)	(0)	(0.03)
	Average ln(Price) reduction for purchases (own brand and branded treated separately)	TPR	−0.016	−0.024	−0.024	−0.137	−0.315	−0.276	−0.372	−0.251	0.077	−0.097	−0.209	−0.442	−0.091	−0.141
		Multibuy	−0.092	−0.100	−0.112	−0.113	−0.372	−0.358	−0.088	−0.254	−0.217	−0.097	0.141	−0.334	−0.144	−0.148
	Percentage increase in per purchase quantity	TPR	**2.9%**	**3.0%**	**3.0%**	**2.3%**	**25.8%**	**4.8%**	**9.2%**	**9.3%**	**13.6%**	**2.6%**	**12.1%**	**2.9%**	**65.6%**	**3.4%**
		Multibuy	**1.9%**	**1.8%**	**1.6%**	**2.3%**	**7.7%**	**22.3%**	**3.5%**	**15.6%**	**0.5%**	**5.8%**	**0.5%**	**1.7%**	**0.5%**	**1.6%**
	Combined percentage increase in combined purchase volume	TPR	**6.8%**	**24.8%**	**27.5%**	**35.4%**	**22.7%**	**20.2%**	**41.3%**	**20.3%**	**42.5%**	**4.4%**	**5.8%**	**22.6%**	**88.6%**	**4.7%**
		Multibuy	**0.8%**	**4.4%**	**5.5%**	**5.2%**	**2.7%**	**32.3%**	**4.2%**	**13.1%**	**0.4%**	**6.5%**	**0.0%**	**3.0%**	**0.3%**	**0.6%**
	Relative effectiveness of TPR versus multibuy		**8.0**	**5.6**	**5.0**	**6.9**	**8.5**	**0.6**	**9.9**	**1.6**	**94.8**	**0.7**	**311.2**	**7.6**	**295.9**	**8.3**

*Note*: The bold is to highlight that those figures are the total impact.

^a^ Not included in calculation of impact because the coefficient is not significant.

The regression results are consistent across all food products in terms of sign and statistical significance for the major variables of interest. The incidence model (Table A4.1) shows that households are more likely to make a purchase when their household inventory is lower and the coefficient for the inclusive value (expected utility of making a purchase) is positive. For all products, the inclusive value (expected utility of making a purchase) has a positive impact on the purchase decision. For consumers of cola, the top quintile and tercile are more responsive to changes in the inclusive value.

In the brand choice, coefficients for price are negative, promotion dummies are positive with brand and product loyalty increasing likelihood of purchases. For all products the TPR dummy coefficient is larger than for multi‐buys: implying that price reductions are more important in both brand choice and in purchase incidence (through the inclusive value). For consumers of cola, the top quintile and tercile are more sensitive to prices and both types of promotion in their product selection than all consumers.

In the quantity decision model, the price coefficient is also negative and coefficients on the promotion dummies are positive. As expected, given that multi‐buy promotions enforce higher purchase volumes, the coefficient for multi‐buy is larger than TPR in the quantity decision.

It is difficult to interpret the coefficients of logit and Poisson models as read. In the literature it is therefore typical to use the derived elasticities and marginal effects the key coefficients of interest. We derive (semi‐) elasticities for key variables in the incidence model (fixed effects logit (Kitazawa, 2012)) and marginal effects for key variables in the quantity model to calculate the aggregate impact of price promotions on purchase volumes (Table 4).

Price promotions manifest in two ways: the price reduction and the indicator (label, color or position) that the product is on promotion. Thus, both the brand choice and purchase quantity models include a dummy for each purchase on promotion and a continuous variable for the price. To calculate the total volume reduction from removing price promotions we use the impact of both the promotion dummy and the price elasticities on the purchase incidence and purchase quantities.

Using the marginal effects at the means calculated from incidence and quantity model (Tables A4.1 and A4.3) we calculate the impact on purchase incidence and quantity for the removal of each type of promotion. For the purchase incidence model the impact of removing price promotions is calculated indirectly through the reduction in the inclusive value from the brand choice model.

### 5.3 Impact on purchase incidence

The removal of promotions leads to a reduction in the average inclusive value for the brand choice (expected value of a purchase). This effect is larger for all products in the case of TPR because of the larger pull in comparison to multi‐buys in the brand selection (Table A4.2). In addition, the average “effective” price reduction associated with TPRs is larger than for multi‐buys for all products except for baked beans where the effective price reduction for a multi‐buy is equivalent to unit price under the multi‐buy promotion.

The overall impact of TPRs on purchase incidence varies from around 4% for everyday biscuits, peanut butter and natural yoghurt to over 30% for lemonade, multipacks of flavored yoghurt, children's biscuits and ketchup. For cola drinks the impact of TPRs is much larger when we consider frequent consumers compared with less frequent.

The effect of removing multi‐buy promotions on incidence is generally smaller than for TPRs, with incidence changes estimated between <1% for children's biscuits, ketchup, peanut butter and natural yoghurt and 23.6% for single large pots of flavored yoghurt. Everyday biscuits and large pots of yoghurt are the only categories for which there is a greater impact of removing multibuys than TPRs. This is likely driven by the frequency of multibuy promotion purchases compared to TPRs: large pot flavored yoghurt are sold on multibuy promotion 36.2% of the time compared to 32.8% TPR, every day biscuits are sold on multibuy promotion 15.0%, compared to 15.3% on TPR.

### 5.4 Impact on purchase quantity

To calculate the impact of removing promotions on purchase quantity conditional on incidence we use the estimated marginal effects of the promotional dummies and the price from the quantity model (Annex 4) and compare to the sales estimate for the average consumer when promotional dummies are set to zero and the price is set to the average non‐promotion price for the products. The reduction in per purchase quantity from removing TPRs varies from 1.3% for lemonade to 13.5% for children's biscuits, while the impact of removing multi‐buys is again smaller for all products, except lemonade, large pots of flavored yoghurt and everyday biscuits, and varies from 0.01% (ketchup and peanut butter) to 25.6% (flavored yoghurt (large pots)).

Once the promotional impacts on purchase incidence and purchase quantity have been combined, we see a larger impact of TPR than multi‐buys on volume purchased for all but two products. For the remaining products, TPR promotions leads to at least 50% greater increases in sales volume than multi‐buy promotions. While it seems counterintuitive that the multi‐buys have a smaller effect on quantity purchased (as inherently the multi‐buy promotion requires a larger quantity of purchase to qualify for the promotion), the relatively lower proportion of this type of promotion in the purchases and the relatively greater percentage price discount in the case of TPRs means that the latter yields a more substantive effect. This impact can be seen most clearly in the Ketchup sub market, as the share of volumes bought on multi‐buy promotion are low (0.2% on multi‐buy vs. 57.3% on TPR). To the contrary, for two products (large pot flavored yoghurt and everyday biscuits) we observed greater effect from removing multi‐buy promotions in comparison to TPRs (13.8% vs. 5.9% for yoghurt, 6.9% vs. 4.6% for everyday biscuits). These products had relatively greater share of multibuy sales compared to TPRs sales.

Our analysis of different subgroups of cola purchasers shows that the removal of both kinds of price promotions are more likely to be impactful on total purchase volumes for more frequent purchasers. We estimate that removing TPRs leads to a 27.3% reduction for the top quintile of cola purchasers, compared to a 6.4% reduction for all cola consumers. In addition, we see that the vast majority of purchase volumes made are consumed by a small number of households. All households purchased 14,242 units while the top tercile purchased 13,330 (93.6%) and the top quintile purchased 12,298 units (86.4%).

The coefficients for key variables are all statistically significant to at least the 1% level. However, a consequence of the reduce form method is that there is no interpretation of, or linkages between, error terms in the sub‐models. The use of a variable that is the predicted value from another regression can be problematic when we consider the estimated standard errors from this regression. However, we are confident that this issue is not substantial given that the *p*‐values for the inclusive value are never over 0.002 in our regression.

## 6 DISCUSSION AND CONCLUSIONS

Price promotions in British supermarkets are not evenly distributed among healthy and unhealthy foods. Sugar sweetened beverages, confectionary and children's cereal are promoted more frequently than dietary staples such as vegetables, grains and fruit. Our analysis of the impact of price promotions on purchases of high‐volume (top tercile) consumers show that both TPRs and multi‐buy promotions encourage additional purchases and greater purchase volumes. For most products, purchases on multibuy promotion are less frequent than on TPRs. For these products, TPRs increase the total purchase volume at least 50% more than multibuys. We find that TPRs encourage more purchases to be made through increasing quantity purchased, and multi‐buys encourage larger quantities to be purchased once the decision to buy has been made, but having a smaller impact on the decision to purchase. Consequently, the removal of either type of price promotions would lead to a lower volume of products purchased. The defining feature of whether TPR is more impactful than multibuy is the relative frequency of each promotion. It follows that policies to restrict price promotions in order to reduce consumption should consider how often each type of promotion occurs.

This paper is the first to use a reduced form demand model with flexible consumption to incorporate household fixed effects and to differentiate between price reduction and multi‐buy promotions. We have found that a specification that allows consumption to depend on inventory fits the data significantly better than one that assumes constant consumption rates. Because consumption is flexible and increases with inventory these additional purchases do not allow consumers to engage in effective, money saving, stockpiling behavior, but lead instead to increased household consumption of that food or drink. This is consistent with marketing literature that finds that price promotions lead to increased consumption (Bell & Boztuğ, 2007; Chan et al., 2008; Ebling & Klapper, 2010; Sun, 2005; Van Heerde et al., 2004; Watt et al., 2020).

Our findings indicate that frequent promotions are leading to greater purchases among high‐volume consumers. We also know that products that are high in sugar, fat and salt are more likely to be promoted and that high consumers of such less healthy products are at greater risk of obesity and related illness (Julia et al., 2015; Mozaffarian, 2016). Therefore, it is reasonable to conclude that price promotions are highly likely to have a negative effect on people's diets (or indeed increase food waste).

Our analysis of households that purchase different quantities of cola drinks indicates that even the top fifth of purchasers of this product are not reaching the point where they are satiated—price promotions contribute to the purchasing of cola for all levels of consumers, and all levels of consumers will drink more cola because of them. In comparing our results, the removal of price promotions is estimated to have a larger relative impact on households that consume greater quantities than for the typical household. Food policy is targeted to reach high consumers of unhealthy food. The ban on price promotions could have broader consequences on the purchasing volumes of infrequent shoppers, however the greatest impact will be on those households consuming the greatest amounts. The top quintile of cola purchasers is responsible for 86% of all cola purchases so the wider impact is likely to be relatively small.

We have elected to analyze ten food groups and two beverages that can be easily stored until needed and (with the exception of yoghurt) do not require refrigeration until they are opened. We estimate that the coefficient of flexible consumption is closer to 1 (less flexible) for ketchup and baked beans than for children's cereal and the sugar sweetened beverages. Our findings are generally in line with the existing literature on price promotions, albeit food categories and products covered in the literature often vary as most of this literature does not consider public health implications. Ailawadi and Neslin (1998) and Ailawadi et al. (2007) found that yoghurt consumption was more flexible than ketchup, Sun (2005) found yoghurt to be more flexible than tuna and Teunter (2002) analyzed six food and drink categories, finding that soft drinks and potato chips had the highest consumption increase through price promotion.

The consumer, when faced with a promotion, may have an expectation that the promotion is for a limited time only, therefore the desire to bring forward future purchases and stockpile is created. Frequent price variation makes food environments harder to navigate, making it harder for shoppers to make informed, healthy decisions about what they buy for themselves and their families. The removal of price promotions^19^ could help improve clarity, however we also need to consider retailer, as well as consumer, response.

While this research is informative about the impact of price promotions in Britain, finding that all types of price promotions lead to increased consumption, it does not allow us to estimate the long run effect of removing price promotions. It does, however, clearly indicate that price promotions are contributing to over‐consumption of some food categories and that price reductions are more impactful than multi‐buys.

Many products that are frequently bought on promotion are high in sugar, salt and fat and are contributing to the obesity epidemic facing the UK. Our results indicate that price promotions are contributing to this, both through multi‐buy promotions but dramatically more so through total price reductions. Policy initiatives to reduce price promotions would be more effective in reducing consumption for frequent consumers of unhealthy food if they were extended to include total price reductions.

## CONFLICTS OF INTEREST

The authors declare no conflict of interest.

## Supporting information

Supporting Information S1

## Data Availability

The data used in this study are from the UK Kantar Fast‐Moving Consumer Goods (FMCG) panel, a nationally representative panel study of food and beverages bought by British households and brought into the home (n≈30,000 per year). Restrictions apply to the availability of these data, which were used under license for this research.

## ENDNOTES

^1^

https://www.oecd.org/els/health‐systems/Obesity‐Update‐2017.pdf

^2^

https://www.gov.uk/government/publications/childhood‐obesity‐a‐plan‐for‐action‐chapter‐2

^3^

https://www.gov.uk/government/news/promotions‐of‐unhealthy‐foods‐restricted‐from‐april‐2022

^4^
In 2012 the Office of Fair Trading (OFT) found price promotions to be anti‐competitive because they reduce price transparency (OFT (2012)), however the precise reason behind the steady fall in promoted sales since 2014 is unknown. There is some speculation that the entry of every‐day low pricing retailers such as Aldi and to the market have enforced consistent and transparent pricing at a permanently lower level, squeezing competitors' margins that leave little room for promotions (Ritson, 2017).

^5^
Consider laundry detergent as an example—stockpiling behavior in order to save money will lead to a higher level of household inventory, but the quantity of detergent you have under your kitchen sink is unlikely to increase the need to wash your clothes; however, an abundance of detergent may lead a household to perform more frequent smaller washes.

^6^
Sainsbury's, Tesco, Morrison, Waitrose, Asda, with a combined market share of 75% in 2017 (https://www.kantarworldpanel.com/en/grocery‐market‐share/great‐britain/snapshot/03.12.17/).

^7^

https://assets.pulishing.service.gov.uk/government/uploads/system/uploads/attachment_data/file/996232/impact‐assessment‐hfss‐advertising.pdf (Table 5).

^8^
We include flavored cereals that are marketed for children: “frosted”, chocolate, honey and cinnamon flavors.

^9^
Crisps sold in single bags are often consumed immediately and would therefore not be suitable for analysis in an inventory model.

^10^
All artificially sweetened, low/no calorie drinks were excluded.

^11^
We exclude single portion, individually sold yoghurts because they are consumed in a single sitting and therefore not suitable analysis in a stockpiling framework.

^12^
Including biscuits such as digestives, chocolate digestives, bourbons, custard creams, packaged cookies, ginger nuts, ginger biscuits, garibaldi, rich tea, oat biscuits, butter biscuits and variety packs.

^13^
The overall results of the model and their implications do not change depending on the unit used.

^14^
Each consumer does not necessarily visit a supermarket every day. We do not model the decision to visit the store, as is typical of this literature: for reasons of tractability, this is assumed to be exogenous.

^15^
This can only be positive if a shopping trip has occurred in *t*−1.

^16^
The‐type depends on the product:

- Baked beans and ketchup we distinguish between standard, low salt, organic, or value
- Peanut butter distinguishes between smooth, crunchy organic, no added salt and alternative nut butter
- Children's cereal we distinguish between types: frosted flakes, honey flakes, plain rice, chocolate rice, chocolate balls, honey balls, honey loops, chocolate loops, sugar puffs, honey puffs, shredded squares, honey shredded squares, frosted shredded squares, chocolate or chocolate orange shredded squares, honey clusters, cookies or chocolate bar imitations.
- Crisps are normal, premium, baked, crinkle cut, light
- Yoghurt types are normal, non‐dairy, diet, high protein, French set, fromage frais
- Everyday biscuits are butter, bourbon, custard cream, digestive, chocolate digestive, cookies, nut, ginger, wafer, shortbread children's biscuits are chocolate, animal, fingers, iced, small cookies, jam, wafer and themed icing where biscuits depict famous characters.

^17^
The packaging distinguishes between cans and large plastic bottles for drinks; plastic, glass or bottles over 1 Kg for ketchup; single or multipacks for baked beans and natural yoghurt; peanut butter, everyday biscuits and children's biscuits are divided into large and small sizes, peanut butter at 500 g, everyday biscuits at 350 g and children's biscuits at 90 g.

^18^
For flavored yoghurt and crisps we distinguish between flavors. For yoghurt these are fruit, fruit combinations, honey, vanilla and chocolate. For crisps there are plain, salt, salt and vinegar, cheese and onion, varieties of meat flavor, prawn cocktail, as well as newer combinations of flavors.

^19^

https://www.gov.uk/government/news/promotions‐of‐unhealthy‐foods‐restricted‐from‐april‐2022

## References

[hec4607-bib-0001] Ailawadi, K. L. , Gedenk, K. , Lutzky, C. , & Neslin, S. A. (2007). Decomposition of the sales impact of promotion‐induced stockpiling. Journal of Marketing Research, 44(3), 450–467. 10.1509/jmkr.44.3.450

[hec4607-bib-0002] Ailawadi, K. L. , & Neslin, S. A. (1998). The effect of promotion on consumption: Buying more and consuming it faster. Journal of Marketing Research, 35(3), 390–398. 10.2307/3152036

[hec4607-bib-0003] Assunção, J. L. , & Meyer, R. J. (1993). The rational effect of price promotions on sales and consumption. Management Science, 39(5), 517–535. 10.1287/mnsc.39.5.517

[hec4607-bib-0005] Bell, D. , & Boztuğ, Y. (2007). The positive and negative effects of inventory on category purchase: An empirical analysis. Marketing Letters, 18(1–2), 1–14. 10.1007/s11002-006-9001-y

[hec4607-bib-0006] Berger, N. , Cummins, S. , Smith, R. D. , & Cornelsen, L. (2019). Recent trends in energy and nutrient content of take‐home food and beverage purchases in Great Britain: An analysis of 225 million food and beverage purchases over 6 years. BMJ Nutrition, Prevention & Health, 2, 63–71. 10.1136/bmjnph-2019-000036 PMC7664498 33235959

[hec4607-bib-0007] Bucklin, R. E. , & Lattin, J. M. (1991). A two‐state model of purchase incidence and brand choice. Marketing Science, 10(1), 24–39. 10.1287/mksc.10.1.24

[hec4607-bib-0008] Chan, T. , Narasimhan, C. , & Zhang, Q. (2008). Decomposing promotional effects with a dynamic structural model of flexible consumption. Journal of Marketing Research, 45(4), 487–498. 10.1509/jmkr.45.4.487

[hec4607-bib-0009] Chandon, P. , & Wansink, B. (2002). When are stockpiled products consumed faster? A convenience‐salience framework of postpurchase consumption incidence and quantity. Journal of Marketing Research, 39(3), 321–335. 10.1509/jmkr.39.3.321.19111

[hec4607-bib-0010] Chandon, P. , & Wansink, B. (2006). How biased household inventory estimates distort shopping and storage decisions. Journal of Marketing, 70(4), 118–135. 10.1509/jmkg.70.4.118

[hec4607-bib-0011] Chandon, P. , & Wansink, B. (2010). Is food marketing making us fat? A multi‐disciplinary review. Foundations and Trends® in Microeconomics, 5(3), 1–86. 10.1561/1700000016

[hec4607-bib-0012] Chintagunta, P. K. (1993). Investigating purchase incidence, brand choice and purchase quantity decisions of households. Marketing Science, 12(2), 184–208. 10.1287/mksc.12.2.184

[hec4607-bib-0013] Commission, U. C. (1999). Supermarkets: A report on the supply of groceries from multiple stores in the United Kingdom. Chapter 7: Pricing.

[hec4607-bib-0004] Competition and Markets Authority . (2008). The groceries market investigation. In C. Commission (Ed.).

[hec4607-bib-0014] Department of Health and Social Care. (2019). Closed consultation: Restricting promotions of food and drink that is high in fat, sugar and salt. Department of Health and Social Care.

[hec4607-bib-0015] Department of Health and Social Care . Childhood obesity: A plan for action, chapter 2 [online]. Department of Health and Social Care. Accessed 25 06 2018. Available: https://www.gov.uk/government/publications/childhood‐obesity‐a‐plan‐for‐action‐chapter‐2

[hec4607-bib-0016] Dobson, P. W. , & Gerstner, E. (2010). For a few cents more: Why supersize unhealthy food? Marketing Science, 29(4), 770–778. 10.1287/mksc.1100.0558

[hec4607-bib-0017] Ebbeling, C. B. , Pawlak, D. B. , & Ludwig, D. S. (2002). Childhood obesity: Public‐health crisis, common sense cure. The Lancet, 360(9331), 473–482. 10.1016/s0140-6736(02)09678-2 12241736

[hec4607-bib-0018] Ebling, C. , & Klapper, D. (2010). Modeling whether, what and how much to buy the right way: An empirical analysis with implications for model building. Review of Managerial Science, 4(3), 171–199. 10.1007/s11846-009-0038-1

[hec4607-bib-0019] Erdem, T. , Imai, S. , & Keane, M. (2003). Brand and quantity choice dynamics under price uncertainty. Quantitative Marketing and Economics, 1, 5–64. 10.1023/a:1023536326497

[hec4607-bib-0020] Fichera, E. , & von Hinke, S. (2020). The response to nutritional labels: Evidence from a quasi‐experiment. Journal of Health Economics, 72, 102326. 10.1016/j.jhealeco.2020.102326 32526549

[hec4607-bib-0021] Folkes, V. , & Wheat, R. D. (1995). Consumers' price perceptions of promoted products. Journal of Retailing, 71(3), 317–328. 10.1016/0022-4359(95)90028-4

[hec4607-bib-0022] Gourville, J. T. , & Soman, D. (1998). Payment depreciation: The behavioral effects of temporally separating payments from consumption. Journal of Consumer Research, 25(2), 160–174. 10.1086/209533

[hec4607-bib-0023] Griffith, R. , von Hinke, S. , & Smith, S. (2018). Getting a healthy start: The effectiveness of targeted benefits for improving dietary choices. Journal of Health Economics, 58, 176–187. 10.1016/j.jhealeco.2018.02.009 29524792 PMC5887873

[hec4607-bib-0024] Gupta, S. (1988). Impact of sales promotions on when, what, and how much to buy. Journal of Marketing Research, 25(4), 342–355. 10.2307/3172945

[hec4607-bib-0025] Hausman, J. , Leonard, G. , & Zona, J. D. (1994). Competitive analysis with differenciated products (pp. 159–180). Annales d'Économie et de Statistique.

[hec4607-bib-0026] Hawkes, C. (2009). Sales promotions and food consumption. Nutrition Reviews, 67(6), 333–342. 10.1111/j.1753-4887.2009.00206.x 19519674

[hec4607-bib-0027] Hendel, I. , & Nevo, A. (2006). Measuring the implications of sales and consumer inventory behavior. Econometrica, 74(6), 1637–1673. 10.1111/j.1468-0262.2006.00721.x

[hec4607-bib-0028] Ho, T.‐H. , Tang, C. S. , & Bell, D. R. (1998). Rational shopping behavior and the option value of variable pricing. Management Science, 44(12‐part‐2), S145–S160. 10.1287/mnsc.44.12.s145

[hec4607-bib-0029] Julia, C. , Ducrot, P. , Lassale, C. , Fézeu, L. , Méjean, C. , Péneau, S. , Touvier, M. , Hercberg, S. , & Kesse‐Guyot, E. (2015). Prospective associations between a dietary index based on the British Food Standard Agency nutrient profiling system and 13‐year weight gain in the SU.VI.MAX cohort. Preventive Medicine, 81, 189–194. 10.1016/j.ypmed.2015.08.022 26348449

[hec4607-bib-0030] Kemp, G. , & Santos Silva, J. (2016). Partial effects in fixed‐effects models. Stata Users Group.

[hec4607-bib-0031] Kitazawa, Y. (2012). Hyperbolic transformation and average elasticity in the framework of the fixed effects logit model. Theroetical Economics Letters, 2(02), 192–199. 10.4236/tel.2012.22034

[hec4607-bib-0032] Mcfadden, D. (1973). Conditional logit analysis of qualitative choice behavior. In P. Zarembka (Ed.), Frontiers in econometrics. Academic Press.

[hec4607-bib-0033] Mozaffarian, D. (2016). Dietary and policy priorities for cardiovascular disease, diabetes, and obesity: A comprehensive review. Circulation, 133(2), 187–225. 10.1161/circulationaha.115.018585 26746178 PMC4814348

[hec4607-bib-0034] Mundlak, Y. (1978). On the pooling of time series and cross section data. Econometrica, 46(1), 69–85. 10.2307/1913646

[hec4607-bib-0035] Nakamura, R. , Suhrcke, M. , Jebb, S. A. , Pechey, R. , Almiron‐Roig, E. , & Marteau, T. M. (2015). Price promotions on healthier compared with less healthy foods: A hierarchical regression analysis of the impact on sales and social patterning of responses to promotions in great Britain. American Journal of Clinical Nutrition, 101(4), 808–816. 10.3945/ajcn.114.094227 25833978 PMC4381774

[hec4607-bib-0036] Neslin, S. A. , & van Heerde, H. J. (2009). Promotion dynamics. Foundations and Trends® in Microeconomics, 3(4), 177–268. 10.1561/1700000010

[hec4607-bib-0037] Pi‐Sunyer, F. X. (1991). Health implications of obesity. The American Journal of Clinical Nutrition, 53(6), 1595S–1603S. 10.1093/ajcn/53.6.1595s 2031492

[hec4607-bib-0038] Raghubir, P. , & Krishna, A. (1999). Vital dimensions in volume perception: Can the eye fool the stomach? Journal of Marketing Research, 36(3), 313–326. 10.2307/3152079

[hec4607-bib-0039] Reilly, J. J. , Methven, E. , Mcdowell, Z. C. , Hacking, B. , Alexander, D. , Stewart, L. , & Kelnar, C. J. H. (2003). Health consequences of obesity. Archives of Disease in Childhood, 88(9), 748–752. 10.1136/adc.88.9.748 12937090 PMC1719633

[hec4607-bib-0040] Ritson . (2017). Is an opinion piece in marketing week. Retrieved from https://www.marketingweek.com/ritson‐era‐price‐promotions‐over/

[hec4607-bib-0041] Santos Silva, J. M. C. (2016). In S. S. Components (Ed.), Aextlogit: Stata module to compute average elasticities for fixed effects logit. Boston College Department of Economics.

[hec4607-bib-0042] Silva‐Risso, J. M. , Bucklin, R. E. , & Morrison, D. G. (1999). A decision support system for planning manufacturers' sales promotion calendars. Marketing Science, 18(3), 274–300. 10.1287/mksc.18.3.274

[hec4607-bib-0043] Smithson, M. , James, K. , & Capelin, C. (2015). Sugar Reduction: The evidence for action Annex 4: An analysis of the role of price promotions. In Public health England. Department of Health and Social Care.

[hec4607-bib-0044] Smyth, C. (2022). Junk food deals U‐turn set to increase food spending. *The Times.* https://www.thetimes.co.uk/article/97521bd8‐d545‐11ec‐bb99‐1bcd45646516?shareToken=1d0d50df63c52fabb61ac5eeae61f13d

[hec4607-bib-0045] Soman, D. A. G. , & John, T. (2001). Transaction decoupling: How price bundling affects the decision to consume. Journal of Marketing Research, 38(1), 30–44. 10.1509/jmkr.38.1.30.18828

[hec4607-bib-0046] Song, I. , & Chintagunta, P. K. (2007). A discrete–continuous model for multicategory purchase behavior of households. Journal of Marketing Research, 44(4), 595–612. 10.1509/jmkr.44.4.595

[hec4607-bib-0047] Statacorp . Asclogit—alternative‐specific conditional logit (Mcfadden’s Choice) Model [Online]. Available: https://www.stata.com/manuals13/rasclogit.pdf. Accessed 13th September 2021.

[hec4607-bib-0048] Sun, B. (2005). Promotion effect on endogenous consumption. Marketing Science, 24(3), 430–443. 10.1287/mksc.1040.0110

[hec4607-bib-0049] Teunter, L. (2002). Analysis of sales promotion effects on household purchase behavior. ERIM PhD Series; EPS‐2002‐016‐ORG (pp. 1–281).

[hec4607-bib-0050] Thomassen, Ø. , Smith, H. , Seiler, S. , & Schiraldi, P. (2017). Multi‐category competition and market power: A model of supermarket pricing. The American Economic Review, 107(8), 2308–2351. 10.1257/aer.20160055

[hec4607-bib-0051] Van Heerde, H. J. , Leeflang, P. S. H. , & Wittink, D. R. (2004). Decomposing the sales promotion bump with store data. Marketing Science, 23(3), 317–334. 10.1287/mksc.1040.0061

[hec4607-bib-0052] Wansink, B. (1996). Can package size accelerate usage volume? Journal of Marketing, 60(3), 1–14. 10.1177/002224299606000301

[hec4607-bib-0053] Wansink, B. , Brasel, S. A. , & Amjad, S. (2000). The mystery of the cabinet castaway: Why we buy products we never use. Journal of Family and Consumer Sciences, 92, 104–108.

[hec4607-bib-0054] Wansink, B. , & Deshpande, R. (1994). 'Out of sight, out of mind': Pantry stockpiling and brand‐usage frequency. Marketing Letters, 5(1), 91–100. 10.1007/bf00993961

[hec4607-bib-0055] Watt, T. L. S. , Beckert, W. , Smith, R. D. , & Cornelsen, L. (2020). Reducing consumption of unhealthy foods and beverages through banning price promotions: What is the evidence and will it work? Public Health Nutrition, 23(12), 1–6. 10.1017/s1368980019004956 32366342 PMC10200664

