# Supplementary material for: The impact of price promotions on sales of unhealthy food and drink products in British retail stores
Source: Health Econ. 2022 Oct 2;32(1):25–46. doi: 10.1002/hec.4607 (PMC10092217; doi:10.1002/hec.4607)
Supplement: Supplementary file 1 — Supporting Information S1 [file HEC-32-25-s001.docx]

# Annex 1: Initialisation period and decision summary statistics

Table A1.1a Summary of households and variables for each household. Values are for the whole year (2017 in the modelling period and 2016 in the initialisation period).

|  | **Cola (all)** | | | | **Cola (top tercile)** | | | | **Cola (top quintile)** | | | | **Lemonade** | | | | **Children's cereal** | | | | **Flavoured Yoghurt (large pots)** | | | | **Flavoured Yoghurt (multi pack)** | | | |
| --- | --- | --- | --- | --- | --- | --- | --- | --- | --- | --- | --- | --- | --- | --- | --- | --- | --- | --- | --- | --- | --- | --- | --- | --- | --- | --- | --- | --- |
|  | **Mean** | **SD** | **Min** | **Max** | **Mean** | **SD** | **Min** | **Max** | **Mean** | **SD** | **Min** | **Max** | **Mean** | **SD** | **Min** | **Max** | **Mean** | **SD** | **Min** | **Max** | **Mean** | **SD** | **Min** | **Max** | **Mean** | **SD** | **Min** | **Max** |
| **Modelling period (2017)** | | | | | | | | | | | | | | | | | | | | | | | | | | | | |
| Purchase incidence^1^ | 0.08 | 0.28 | 0.00 | 1.00 | 0.14 | 0.35 | 0.00 | 1.00 | 0.19 | 0.39 | 0.00 | 1.00 | 0.10 | 0.30 | 0.00 | 1.00 | 0.11 | 0.31 | 0.00 | 1.00 | 0.12 | 0.33 | 0.00 | 1.00 | 0.18 | 0.38 | 0.00 | 1.00 |
| # of packs per incidence | 1.45 | 0.94 | 1.00 | 16.00 | 1.50 | 0.99 | 1.00 | 16.00 | 1.56 | 1.04 | 1.00 | 16.00 | 1.48 | 0.86 | 1.00 | 17.00 | 1.26 | 0.59 | 1.00 | 10.00 | 1.72 | 0.87 | 1.00 | 13.00 | 1.53 | 0.86 | 1.00 | 12.00 |
| Price (£/Lt or £/Kg) | 0.71 | 0.28 | 0.17 | 1.64 | 0.70 | 0.28 | 0.17 | 1.64 | 0.68 | 0.28 | 0.17 | 1.61 | 0.41 | 0.28 | 0.10 | 2.22 | 3.78 | 1.13 | 0.52 | 10.35 | 2.52 | 0.59 | 0.30 | 7.08 | 2.18 | 1.02 | 0.33 | 7.25 |
| **Initialisation period (2016)** | | | | | | | | | | | | | | | | | | | | | | | | | | | | |
| Av. Consumption (Cl)^5^ | 8.65 | 14.77 | 0.34 | 167.84 | 15.50 | 18.56 | 0.34 | 167.84 | 21.57 | 21.15 | 0.55 | 167.84 | 7.73 | 10.58 | 0.55 | 139.36 | 2.33 | 1.88 | 0.11 | 20.39 | 2.03 | 2.62 | 0.12 | 27.27 | 4.01 | 4.35 | 0.12 | 32.96 |
| Brand Loyalty 1 | 0.35 | 0.44 | 0.00 | 1.00 | 0.41 | 0.45 | 0.00 | 1.00 | 0.46 | 0.46 | 0.00 | 1.00 | 0.02 | 0.12 | 0.00 | 1.00 | 0.50 | 0.37 | 0.00 | 1.00 | 0.15 | 0.29 | 0.00 | 1.00 | 0.28 | 0.40 | 0.00 | 1.00 |
| Brand Loyalty 2 | 0.50 | 0.47 | 0.00 | 1.00 | 0.42 | 0.45 | 0.00 | 1.00 | 0.36 | 0.44 | 0.00 | 1.00 | 0.04 | 0.15 | 0.00 | 1.00 | 0.33 | 0.35 | 0.00 | 1.00 | 0.11 | 0.29 | 0.00 | 1.00 | 0.13 | 0.29 | 0.00 | 1.00 |
| Brand Loyalty 3 | 0.15 | 0.34 | 0.00 | 1.00 | 0.17 | 0.35 | 0.00 | 1.00 | 0.18 | 0.37 | 0.00 | 1.00 | 0.17 | 0.32 | 0.00 | 1.00 | 0.10 | 0.24 | 0.00 | 1.00 | 0.06 | 0.20 | 0.00 | 1.00 | 0.01 | 0.06 | 0.00 | 1.00 |
| Brand Loyalty 4 |  |  |  |  |  |  |  |  |  |  |  |  | 0.05 | 0.15 | 0.00 | 1.00 | 0.01 | 0.06 | 0.00 | 1.00 | 0.06 | 0.20 | 0.00 | 1.00 | 0.02 | 0.10 | 0.00 | 1.00 |
| Brand Loyalty 5 |  |  |  |  |  |  |  |  |  |  |  |  | 0.09 | 0.22 | 0.00 | 1.00 | 0.05 | 0.16 | 0.00 | 1.00 | 0.00 | 0.00 | 0.00 | 0.00 | 0.00 | 0.03 | 0.00 | 0.73 |
| Brand Loyalty 6 |  |  |  |  |  |  |  |  |  |  |  |  | 0.07 | 0.20 | 0.00 | 1.00 |  |  |  |  | 0.01 | 0.07 | 0.00 | 1.00 | 0.45 | 0.43 | 0.00 | 1.00 |
| Brand Loyalty 7 |  |  |  |  |  |  |  |  |  |  |  |  | 0.02 | 0.12 | 0.00 | 1.00 |  |  |  |  | 0.30 | 0.38 | 0.00 | 1.00 | 0.01 | 0.06 | 0.00 | 1.00 |
| Brand Loyalty 8 |  |  |  |  |  |  |  |  |  |  |  |  | 0.03 | 0.14 | 0.00 | 1.00 |  |  |  |  | 0.06 | 0.16 | 0.00 | 1.00 | 0.01 | 0.05 | 0.00 | 0.80 |
| Brand Loyalty 9 |  |  |  |  |  |  |  |  |  |  |  |  | 0.52 | 0.44 | 0.00 | 1.00 |  |  |  |  | 0.26 | 0.35 | 0.00 | 1.00 | 0.03 | 0.12 | 0.00 | 1.00 |
| Brand Loyalty 10 |  |  |  |  |  |  |  |  |  |  |  |  |  |  |  |  |  |  |  |  |  |  |  |  | 0.03 | 0.14 | 0.00 | 1.00 |
| Brand Loyalty 11 |  |  |  |  |  |  |  |  |  |  |  |  |  |  |  |  |  |  |  |  |  |  |  |  | 0.01 | 0.10 | 0.00 | 1.00 |
| Brand Loyalty 12 |  |  |  |  |  |  |  |  |  |  |  |  |  |  |  |  |  |  |  |  |  |  |  |  | 0.02 | 0.11 | 0.00 | 1.00 |
| Packaging loyalty 1 | 0.51 | 0.47 | 0.00 | 1.00 | 0.55 | 0.46 | 0.00 | 1.00 | 0.60 | 0.45 | 0.00 | 1.00 | 0.91 | 0.24 | 0.00 | 1.00 |  |  |  |  |  |  |  |  |  |  |  |  |
| Packaging loyalty 2 | 0.49 | 0.47 | 0.00 | 1.00 | 0.45 | 0.46 | 0.00 | 1.00 | 0.40 | 0.45 | 0.00 | 1.00 | 0.09 | 0.24 | 0.00 | 1.00 |  |  |  |  |  |  |  |  |  |  |  |  |
| Type loyalty 1 |  |  |  |  |  |  |  |  |  |  |  |  |  |  |  |  | 0.00 | 0.01 | 0.00 | 0.28 | 0.03 | 0.13 | 0.00 | 1.00 | 0.04 | 0.16 | 0.00 | 1.00 |
| Type loyalty 2 |  |  |  |  |  |  |  |  |  |  |  |  |  |  |  |  | 0.05 | 0.15 | 0.00 | 1.00 | 0.34 | 0.38 | 0.00 | 1.00 | 0.01 | 0.06 | 0.00 | 1.00 |
| Type loyalty 3 |  |  |  |  |  |  |  |  |  |  |  |  |  |  |  |  | 0.16 | 0.26 | 0.00 | 1.00 | 0.08 | 0.22 | 0.00 | 1.00 | 0.04 | 0.14 | 0.00 | 1.00 |
| Type loyalty 4 |  |  |  |  |  |  |  |  |  |  |  |  |  |  |  |  | 0.01 | 0.05 | 0.00 | 1.00 | 0.03 | 0.14 | 0.00 | 1.00 | 0.06 | 0.18 | 0.00 | 1.00 |
| Type loyalty 5 |  |  |  |  |  |  |  |  |  |  |  |  |  |  |  |  | 0.04 | 0.13 | 0.00 | 1.00 | 0.11 | 0.29 | 0.00 | 1.00 | 0.24 | 0.35 | 0.00 | 1.00 |
| Type loyalty 6 |  |  |  |  |  |  |  |  |  |  |  |  |  |  |  |  | 0.05 | 0.17 | 0.00 | 1.00 | 0.41 | 0.39 | 0.00 | 1.00 | 0.01 | 0.06 | 0.00 | 1.00 |
| Type loyalty 7 |  |  |  |  |  |  |  |  |  |  |  |  |  |  |  |  | 0.03 | 0.10 | 0.00 | 1.00 |  |  |  |  | 0.59 | 0.41 | 0.00 | 1.00 |
| Type loyalty 8 |  |  |  |  |  |  |  |  |  |  |  |  |  |  |  |  | 0.12 | 0.24 | 0.00 | 1.00 |  |  |  |  | 0.02 | 0.10 | 0.00 | 1.00 |
| Type loyalty 9 |  |  |  |  |  |  |  |  |  |  |  |  |  |  |  |  | 0.04 | 0.14 | 0.00 | 1.00 |  |  |  |  | 0.00 | 0.05 | 0.00 | 1.00 |
| Type loyalty 10 |  |  |  |  |  |  |  |  |  |  |  |  |  |  |  |  | 0.01 | 0.04 | 0.00 | 0.93 |  |  |  |  |  |  |  |  |
| Type loyalty 11 |  |  |  |  |  |  |  |  |  |  |  |  |  |  |  |  | 0.15 | 0.28 | 0.00 | 1.00 |  |  |  |  |  |  |  |  |
| Type loyalty 12 |  |  |  |  |  |  |  |  |  |  |  |  |  |  |  |  | 0.04 | 0.14 | 0.00 | 1.00 |  |  |  |  |  |  |  |  |
| Type loyalty 13 |  |  |  |  |  |  |  |  |  |  |  |  |  |  |  |  | 0.01 | 0.07 | 0.00 | 1.00 |  |  |  |  |  |  |  |  |
| Type loyalty 14 |  |  |  |  |  |  |  |  |  |  |  |  |  |  |  |  | 0.01 | 0.06 | 0.00 | 1.00 |  |  |  |  |  |  |  |  |
| Type loyalty 15 |  |  |  |  |  |  |  |  |  |  |  |  |  |  |  |  | 0.12 | 0.25 | 0.00 | 1.00 |  |  |  |  |  |  |  |  |
| Type loyalty 16 |  |  |  |  |  |  |  |  |  |  |  |  |  |  |  |  | 0.15 | 0.27 | 0.00 | 1.00 |  |  |  |  |  |  |  |  |
| Type loyalty 17 |  |  |  |  |  |  |  |  |  |  |  |  |  |  |  |  | 0.01 | 0.06 | 0.00 | 1.00 |  |  |  |  |  |  |  |  |
| Flavour loyalty 1 |  |  |  |  |  |  |  |  |  |  |  |  |  |  |  |  |  |  |  |  | 0.02 | 0.11 | 0.00 | 1.00 | 0.28 | 0.33 | 0.00 | 1.00 |
| Flavour loyalty 2 |  |  |  |  |  |  |  |  |  |  |  |  |  |  |  |  |  |  |  |  | 0.00 | 0.02 | 0.00 | 0.50 | 0.05 | 0.15 | 0.00 | 1.00 |
| Flavour loyalty 3 |  |  |  |  |  |  |  |  |  |  |  |  |  |  |  |  |  |  |  |  | 0.01 | 0.08 | 0.00 | 1.00 | 0.06 | 0.18 | 0.00 | 1.00 |
| Flavour loyalty 4 |  |  |  |  |  |  |  |  |  |  |  |  |  |  |  |  |  |  |  |  | 0.03 | 0.11 | 0.00 | 1.00 | 0.01 | 0.07 | 0.00 | 1.00 |
| Flavour loyalty 5 |  |  |  |  |  |  |  |  |  |  |  |  |  |  |  |  |  |  |  |  | 0.00 | 0.00 | 0.00 | 0.00 | 0.19 | 0.29 | 0.00 | 1.00 |
| Flavour loyalty 6 |  |  |  |  |  |  |  |  |  |  |  |  |  |  |  |  |  |  |  |  | 0.01 | 0.06 | 0.00 | 1.00 | 0.03 | 0.13 | 0.00 | 1.00 |
| Flavour loyalty 7 |  |  |  |  |  |  |  |  |  |  |  |  |  |  |  |  |  |  |  |  | 0.05 | 0.14 | 0.00 | 1.00 | 0.00 | 0.02 | 0.00 | 0.57 |
| Flavour loyalty 8 |  |  |  |  |  |  |  |  |  |  |  |  |  |  |  |  |  |  |  |  | 0.05 | 0.15 | 0.00 | 1.00 | 0.01 | 0.08 | 0.00 | 0.88 |
| Flavour loyalty 9 |  |  |  |  |  |  |  |  |  |  |  |  |  |  |  |  |  |  |  |  | 0.06 | 0.20 | 0.00 | 1.00 | 0.02 | 0.11 | 0.00 | 1.00 |
| Flavour loyalty 10 |  |  |  |  |  |  |  |  |  |  |  |  |  |  |  |  |  |  |  |  | 0.00 | 0.05 | 0.00 | 1.00 | 0.03 | 0.12 | 0.00 | 1.00 |
| Flavour loyalty 11 |  |  |  |  |  |  |  |  |  |  |  |  |  |  |  |  |  |  |  |  | 0.00 | 0.04 | 0.00 | 1.00 | 0.01 | 0.06 | 0.00 | 1.00 |
| Flavour loyalty 12 |  |  |  |  |  |  |  |  |  |  |  |  |  |  |  |  |  |  |  |  | 0.01 | 0.05 | 0.00 | 0.72 | 0.03 | 0.12 | 0.00 | 1.00 |
| Flavour loyalty 13 |  |  |  |  |  |  |  |  |  |  |  |  |  |  |  |  |  |  |  |  | 0.01 | 0.04 | 0.00 | 0.75 | 0.01 | 0.05 | 0.00 | 1.00 |
| Flavour loyalty 14 |  |  |  |  |  |  |  |  |  |  |  |  |  |  |  |  |  |  |  |  | 0.11 | 0.27 | 0.00 | 1.00 | 0.02 | 0.11 | 0.00 | 1.00 |
| Flavour loyalty 15 |  |  |  |  |  |  |  |  |  |  |  |  |  |  |  |  |  |  |  |  | 0.06 | 0.17 | 0.00 | 1.00 | 0.01 | 0.06 | 0.00 | 1.00 |
| Flavour loyalty 16 |  |  |  |  |  |  |  |  |  |  |  |  |  |  |  |  |  |  |  |  | 0.01 | 0.05 | 0.00 | 0.79 | 0.01 | 0.08 | 0.00 | 1.00 |
| Flavour loyalty 17 |  |  |  |  |  |  |  |  |  |  |  |  |  |  |  |  |  |  |  |  | 0.01 | 0.08 | 0.00 | 1.00 | 0.02 | 0.07 | 0.00 | 1.00 |
| Flavour loyalty 18 |  |  |  |  |  |  |  |  |  |  |  |  |  |  |  |  |  |  |  |  | 0.07 | 0.19 | 0.00 | 1.00 | 0.01 | 0.09 | 0.00 | 1.00 |
| Flavour loyalty 19 |  |  |  |  |  |  |  |  |  |  |  |  |  |  |  |  |  |  |  |  | 0.00 | 0.00 | 0.00 | 0.00 | 0.09 | 0.22 | 0.00 | 1.00 |
| Flavour loyalty 20 |  |  |  |  |  |  |  |  |  |  |  |  |  |  |  |  |  |  |  |  | 0.00 | 0.02 | 0.00 | 0.50 | 0.00 | 0.00 | 0.00 | 0.00 |
| Flavour loyalty 21 |  |  |  |  |  |  |  |  |  |  |  |  |  |  |  |  |  |  |  |  | 0.01 | 0.07 | 0.00 | 1.00 | 0.02 | 0.11 | 0.00 | 1.00 |
| Flavour loyalty 22 |  |  |  |  |  |  |  |  |  |  |  |  |  |  |  |  |  |  |  |  | 0.00 | 0.00 | 0.00 | 0.00 | 0.04 | 0.16 | 0.00 | 1.00 |
| Flavour loyalty 23 |  |  |  |  |  |  |  |  |  |  |  |  |  |  |  |  |  |  |  |  | 0.01 | 0.07 | 0.00 | 1.00 | 0.02 | 0.13 | 0.00 | 1.00 |
| Flavour loyalty 24 |  |  |  |  |  |  |  |  |  |  |  |  |  |  |  |  |  |  |  |  | 0.00 | 0.02 | 0.00 | 0.33 | 0.00 | 0.04 | 0.00 | 0.80 |
| Flavour loyalty 25 |  |  |  |  |  |  |  |  |  |  |  |  |  |  |  |  |  |  |  |  | 0.00 | 0.04 | 0.00 | 0.57 | 0.03 | 0.12 | 0.00 | 0.93 |
| Flavour loyalty 26 |  |  |  |  |  |  |  |  |  |  |  |  |  |  |  |  |  |  |  |  | 0.01 | 0.05 | 0.00 | 1.00 |  |  |  |  |
| Flavour loyalty 27 |  |  |  |  |  |  |  |  |  |  |  |  |  |  |  |  |  |  |  |  | 0.05 | 0.15 | 0.00 | 1.00 |  |  |  |  |
| Flavour loyalty 28 |  |  |  |  |  |  |  |  |  |  |  |  |  |  |  |  |  |  |  |  | 0.02 | 0.09 | 0.00 | 1.00 |  |  |  |  |
| Flavour loyalty 29 |  |  |  |  |  |  |  |  |  |  |  |  |  |  |  |  |  |  |  |  | 0.18 | 0.30 | 0.00 | 1.00 |  |  |  |  |
| Flavour loyalty 30 |  |  |  |  |  |  |  |  |  |  |  |  |  |  |  |  |  |  |  |  | 0.01 | 0.08 | 0.00 | 1.00 |  |  |  |  |
| Flavour loyalty 31 |  |  |  |  |  |  |  |  |  |  |  |  |  |  |  |  |  |  |  |  | 0.00 | 0.00 | 0.00 | 0.00 |  |  |  |  |
| Flavour loyalty 32 |  |  |  |  |  |  |  |  |  |  |  |  |  |  |  |  |  |  |  |  | 0.14 | 0.28 | 0.00 | 1.00 |  |  |  |  |
| Flavour loyalty 33 |  |  |  |  |  |  |  |  |  |  |  |  |  |  |  |  |  |  |  |  | 0.00 | 0.00 | 0.00 | 0.00 |  |  |  |  |
| Flavour loyalty 34 |  |  |  |  |  |  |  |  |  |  |  |  |  |  |  |  |  |  |  |  | 0.02 | 0.08 | 0.00 | 0.94 |  |  |  |  |
| Flavour loyalty 35 |  |  |  |  |  |  |  |  |  |  |  |  |  |  |  |  |  |  |  |  | 0.02 | 0.10 | 0.00 | 0.88 |  |  |  |  |

*Notes: For each household: 1. The % of store visits that result in a purchase of the good in question. 2. The number of packs bought. 3. The average daily consumption per household in centilitres in the initialisation period. 4. A [0,1] variable indicating loyalty to brand by share of volume purchased in initialisation period. 5. Same loyalty calculation for package type. 6. Same loyalty calculation for type of product.*

Table A1.1b Summary of households and variables for each household. Values are for the whole year (2017 in the modelling period and 2016 in the initialisation period).

|  | **Crisps (multi pack)** | | | | **Children's biscuits** | | | | **Everyday Biscuits** | | | | **Peanut butter** | | | | **Baked beans** | | | | **Ketchup** | | | | **Natural Yoghurt** | | | |
| --- | --- | --- | --- | --- | --- | --- | --- | --- | --- | --- | --- | --- | --- | --- | --- | --- | --- | --- | --- | --- | --- | --- | --- | --- | --- | --- | --- | --- |
|  | **Mean** | **SD** | **Min** | **Max** | **Mean** | **SD** | **Min** | **Max** | **Mean** | **SD** | **Min** | **Max** | **Mean** | **SD** | **Min** | **Max** | **Mean** | **SD** | **Min** | **Max** | **Mean** | **SD** | **Min** | **Max** | **Mean** | **SD** | **Min** | **Max** |
| **Modelling period (2017)** | | | | | | | | | | | | | | | | | | | | | | | | | | | | |
| Purchase incidence^1^ | 0.16 | 0.37 | 0.00 | 1.00 | 0.13 | 0.33 | 0.00 | 1.00 | 0.15 | 0.36 | 0.00 | 1.00 | 0.08 | 0.27 | 0.00 | 1.00 | 0.15 | 0.36 | 0.00 | 1.00 | 0.07 | 0.25 | 0.00 | 1.00 | 0.19 | 0.39 | 0.00 | 1.00 |
| # of packs per incidence | 1.56 | 0.80 | 1.00 | 10.00 | 1.59 | 0.94 | 1.00 | 15.00 | 1.71 | 1.13 | 1.00 | 26.00 | 1.15 | 0.53 | 1.00 | 12.00 | 1.65 | 1.13 | 1.00 | 20.00 | 1.06 | 0.28 | 1.00 | 8.00 | 1.38 | 0.83 | 1.00 | 12.00 |
| Price (£/Lt or £/Kg) | 7.09 | 2.02 | 1.07 | 22.67 | 6.50 | 2.23 | 0.68 | 35.19 | 1.90 | 0.87 | 0.70 | 8.00 | 3.98 | 1.87 | 1.53 | 14.10 | 1.16 | 0.71 | 0.12 | 7.26 | 2.66 | 0.71 | 0.55 | 7.69 | 2.43 | 1.13 | 0.33 | 16.18 |
| **Initialisation period (2016)** | | | | | | | | | | | | | | | | | | | | | | | | | | | | |
| Av. Consumption (Cl)^5^ | 0.94 | 0.88 | 0.04 | 9.83 | 0.68 | 0.69 | 0.02 | 9.32 | 1.92 | 2.03 | 0.06 | 22.44 | 0.90 | 0.79 | 0.09 | 8.43 | 4.20 | 3.39 | 0.17 | 50.10 | 1.20 | 1.04 | 0.09 | 15.90 | 3.00 | 3.44 | 0.03 | 51.19 |
| Brand Loyalty 1 | 0.19 | 0.34 | 0.00 | 1.00 | 0.03 | 0.12 | 0.00 | 1.00 | 0.69 | 0.36 | 0.00 | 1.00 | 0.61 | 0.43 | 0.00 | 1.00 | 0.15 | 0.31 | 0.00 | 1.00 | 0.78 | 0.38 | 0.00 | 1.00 | 0.59 | 0.42 | 0.00 | 1.00 |
| Brand Loyalty 2 | 0.01 | 0.08 | 0.00 | 1.00 | 0.00 | 0.05 | 0.00 | 1.00 | 0.01 | 0.06 | 0.00 | 1.00 | 0.00 | 0.03 | 0.00 | 0.75 | 0.38 | 0.44 | 0.00 | 1.00 | 0.02 | 0.12 | 0.00 | 1.00 | 0.00 | 0.05 | 0.00 | 0.95 |
| Brand Loyalty 3 | 0.00 | 0.03 | 0.00 | 0.55 | 0.12 | 0.24 | 0.00 | 1.00 | 0.01 | 0.06 | 0.00 | 1.00 | 0.00 | 0.00 | 0.00 | 0.00 | 0.46 | 0.46 | 0.00 | 1.00 | 0.20 | 0.37 | 0.00 | 1.00 | 0.03 | 0.15 | 0.00 | 1.00 |
| Brand Loyalty 4 | 0.07 | 0.19 | 0.00 | 1.00 | 0.24 | 0.32 | 0.00 | 1.00 | 0.01 | 0.08 | 0.00 | 1.00 | 0.00 | 0.01 | 0.00 | 0.22 | 0.01 | 0.08 | 0.00 | 1.00 |  |  |  |  | 0.03 | 0.13 | 0.00 | 1.00 |
| Brand Loyalty 5 | 0.73 | 0.38 | 0.00 | 1.00 | 0.09 | 0.18 | 0.00 | 1.00 | 0.01 | 0.07 | 0.00 | 1.00 | 0.05 | 0.19 | 0.00 | 1.00 |  |  |  |  |  |  |  |  | 0.00 | 0.04 | 0.00 | 1.00 |
| Brand Loyalty 6 |  |  |  |  | 0.00 | 0.00 | 0.00 | 0.00 | 0.24 | 0.35 | 0.00 | 1.00 | 0.00 | 0.03 | 0.00 | 0.75 |  |  |  |  |  |  |  |  | 0.00 | 0.02 | 0.00 | 0.37 |
| Brand Loyalty 7 |  |  |  |  | 0.04 | 0.15 | 0.00 | 1.00 | 0.02 | 0.10 | 0.00 | 1.00 | 0.00 | 0.02 | 0.00 | 0.47 |  |  |  |  |  |  |  |  | 0.01 | 0.04 | 0.00 | 0.85 |
| Brand Loyalty 8 |  |  |  |  | 0.01 | 0.05 | 0.00 | 0.96 |  |  |  |  | 0.00 | 0.02 | 0.00 | 0.60 |  |  |  |  |  |  |  |  | 0.00 | 0.03 | 0.00 | 0.93 |
| Brand Loyalty 9 |  |  |  |  | 0.13 | 0.21 | 0.00 | 1.00 |  |  |  |  | 0.01 | 0.07 | 0.00 | 1.00 |  |  |  |  |  |  |  |  | 0.00 | 0.01 | 0.00 | 0.24 |
| Brand Loyalty 10 |  |  |  |  | 0.00 | 0.00 | 0.00 | 0.06 |  |  |  |  | 0.00 | 0.02 | 0.00 | 0.50 |  |  |  |  |  |  |  |  | 0.06 | 0.20 | 0.00 | 1.00 |
| Brand Loyalty 11 |  |  |  |  | 0.00 | 0.03 | 0.00 | 0.55 |  |  |  |  | 0.00 | 0.01 | 0.00 | 0.41 |  |  |  |  |  |  |  |  | 0.01 | 0.05 | 0.00 | 1.00 |
| Brand Loyalty 12 |  |  |  |  | 0.00 | 0.03 | 0.00 | 1.00 |  |  |  |  | 0.18 | 0.33 | 0.00 | 1.00 |  |  |  |  |  |  |  |  | 0.00 | 0.04 | 0.00 | 1.00 |
| Brand Loyalty 13 |  |  |  |  | 0.00 | 0.03 | 0.00 | 1.00 |  |  |  |  | 0.14 | 0.30 | 0.00 | 1.00 |  |  |  |  |  |  |  |  | 0.00 | 0.00 | 0.00 | 0.00 |
| Brand Loyalty 14 |  |  |  |  | 0.01 | 0.06 | 0.00 | 1.00 |  |  |  |  |  |  |  |  |  |  |  |  |  |  |  |  | 0.04 | 0.16 | 0.00 | 1.00 |
| Brand Loyalty 15 |  |  |  |  | 0.09 | 0.19 | 0.00 | 1.00 |  |  |  |  |  |  |  |  |  |  |  |  |  |  |  |  | 0.00 | 0.05 | 0.00 | 1.00 |
| Brand Loyalty 16 |  |  |  |  | 0.16 | 0.24 | 0.00 | 1.00 |  |  |  |  |  |  |  |  |  |  |  |  |  |  |  |  | 0.01 | 0.06 | 0.00 | 1.00 |
| Brand Loyalty 17 |  |  |  |  | 0.01 | 0.06 | 0.00 | 1.00 |  |  |  |  |  |  |  |  |  |  |  |  |  |  |  |  | 0.01 | 0.06 | 0.00 | 1.00 |
| Brand Loyalty 18 |  |  |  |  | 0.00 | 0.04 | 0.00 | 1.00 |  |  |  |  |  |  |  |  |  |  |  |  |  |  |  |  | 0.00 | 0.02 | 0.00 | 1.00 |
| Brand Loyalty 19 |  |  |  |  | 0.00 | 0.01 | 0.00 | 0.30 |  |  |  |  |  |  |  |  |  |  |  |  |  |  |  |  | 0.00 | 0.01 | 0.00 | 0.24 |
| Brand Loyalty 20 |  |  |  |  | 0.02 | 0.08 | 0.00 | 1.00 |  |  |  |  |  |  |  |  |  |  |  |  |  |  |  |  | 0.09 | 0.25 | 0.00 | 1.00 |
| Brand Loyalty 21 |  |  |  |  | 0.04 | 0.13 | 0.00 | 1.00 |  |  |  |  |  |  |  |  |  |  |  |  |  |  |  |  | 0.00 | 0.02 | 0.00 | 0.69 |
| Brand Loyalty 22 |  |  |  |  | 0.00 | 0.00 | 0.00 | 0.07 |  |  |  |  |  |  |  |  |  |  |  |  |  |  |  |  | 0.11 | 0.27 | 0.00 | 1.00 |
| Packaging loyalty 1 |  |  |  |  | 0.99 | 0.08 | 0.00 | 1.00 | 0.42 | 0.35 | 0.00 | 1.00 | 0.25 | 0.36 | 0.00 | 1.00 | 0.43 | 0.40 | 0.00 | 1.00 | 0.06 | 0.18 | 0.00 | 1.00 | 0.97 | 0.12 | 0.00 | 1.00 |
| Packaging loyalty 2 |  |  |  |  | 0.01 | 0.08 | 0.00 | 1.00 | 0.58 | 0.35 | 0.00 | 1.00 | 0.75 | 0.36 | 0.00 | 1.00 | 0.57 | 0.40 | 0.00 | 1.00 | 0.02 | 0.12 | 0.00 | 1.00 | 0.03 | 0.12 | 0.00 | 1.00 |
| Packaging loyalty 3 |  |  |  |  |  |  |  |  |  |  |  |  |  |  |  |  |  |  |  |  | 0.92 | 0.22 | 0.00 | 1.00 |  |  |  |  |
| Type loyalty 1 | 0.06 | 0.19 | 0.00 | 1.00 | 0.07 | 0.15 | 0.00 | 1.00 | 0.05 | 0.15 | 0.00 | 1.00 | 0.60 | 0.42 | 0.00 | 1.00 | 0.01 | 0.08 | 0.00 | 1.00 | 0.02 | 0.12 | 0.00 | 1.00 | 0.15 | 0.27 | 0.00 | 1.00 |
| Type loyalty 2 | 0.09 | 0.23 | 0.00 | 1.00 | 0.00 | 0.02 | 0.00 | 0.39 | 0.03 | 0.10 | 0.00 | 1.00 | 0.02 | 0.08 | 0.00 | 0.91 | 0.09 | 0.25 | 0.00 | 1.00 | 0.95 | 0.20 | 0.00 | 1.00 | 0.01 | 0.06 | 0.00 | 1.00 |
| Type loyalty 3 | 0.02 | 0.12 | 0.00 | 1.00 | 0.02 | 0.09 | 0.00 | 1.00 | 0.00 | 0.00 | 0.00 | 0.00 | 0.01 | 0.07 | 0.00 | 1.00 | 0.00 | 0.06 | 0.00 | 1.00 | 0.03 | 0.16 | 0.00 | 1.00 | 0.27 | 0.36 | 0.00 | 1.00 |
| Type loyalty 4 | 0.81 | 0.31 | 0.00 | 1.00 | 0.09 | 0.19 | 0.00 | 1.00 | 0.06 | 0.18 | 0.00 | 1.00 | 0.02 | 0.10 | 0.00 | 1.00 | 0.70 | 0.40 | 0.00 | 1.00 |  |  |  |  | 0.19 | 0.31 | 0.00 | 1.00 |
| Type loyalty 5 | 0.01 | 0.09 | 0.00 | 1.00 | 0.04 | 0.12 | 0.00 | 1.00 | 0.09 | 0.19 | 0.00 | 1.00 | 0.01 | 0.06 | 0.00 | 1.00 | 0.19 | 0.36 | 0.00 | 1.00 |  |  |  |  | 0.03 | 0.16 | 0.00 | 1.00 |
| Type loyalty 6 |  |  |  |  | 0.02 | 0.09 | 0.00 | 1.00 | 0.27 | 0.33 | 0.00 | 1.00 | 0.00 | 0.04 | 0.00 | 0.67 |  |  |  |  |  |  |  |  | 0.32 | 0.38 | 0.00 | 1.00 |
| Type loyalty 7 |  |  |  |  | 0.11 | 0.20 | 0.00 | 1.00 | 0.03 | 0.13 | 0.00 | 1.00 | 0.01 | 0.08 | 0.00 | 1.00 |  |  |  |  |  |  |  |  | 0.03 | 0.14 | 0.00 | 1.00 |
| Type loyalty 8 |  |  |  |  | 0.01 | 0.07 | 0.00 | 1.00 | 0.04 | 0.13 | 0.00 | 1.00 | 0.02 | 0.11 | 0.00 | 1.00 |  |  |  |  |  |  |  |  |  |  |  |  |
| Type loyalty 9 |  |  |  |  | 0.03 | 0.10 | 0.00 | 1.00 | 0.09 | 0.20 | 0.00 | 1.00 | 0.31 | 0.41 | 0.00 | 1.00 |  |  |  |  |  |  |  |  |  |  |  |  |
| Type loyalty 10 |  |  |  |  | 0.11 | 0.20 | 0.00 | 1.00 | 0.04 | 0.15 | 0.00 | 1.00 |  |  |  |  |  |  |  |  |  |  |  |  |  |  |  |  |
| Type loyalty 11 |  |  |  |  | 0.05 | 0.14 | 0.00 | 1.00 | 0.08 | 0.19 | 0.00 | 1.00 |  |  |  |  |  |  |  |  |  |  |  |  |  |  |  |  |
| Type loyalty 12 |  |  |  |  | 0.23 | 0.31 | 0.00 | 1.00 | 0.01 | 0.05 | 0.00 | 0.83 |  |  |  |  |  |  |  |  |  |  |  |  |  |  |  |  |
| Type loyalty 13 |  |  |  |  | 0.08 | 0.17 | 0.00 | 1.00 | 0.01 | 0.08 | 0.00 | 1.00 |  |  |  |  |  |  |  |  |  |  |  |  |  |  |  |  |
| Type loyalty 14 |  |  |  |  | 0.00 | 0.00 | 0.00 | 0.14 | 0.13 | 0.25 | 0.00 | 1.00 |  |  |  |  |  |  |  |  |  |  |  |  |  |  |  |  |
| Type loyalty 15 |  |  |  |  | 0.02 | 0.11 | 0.00 | 1.00 | 0.01 | 0.08 | 0.00 | 1.00 |  |  |  |  |  |  |  |  |  |  |  |  |  |  |  |  |
| Type loyalty 16 |  |  |  |  | 0.02 | 0.07 | 0.00 | 1.00 | 0.01 | 0.06 | 0.00 | 0.91 |  |  |  |  |  |  |  |  |  |  |  |  |  |  |  |  |
| Type loyalty 17 |  |  |  |  | 0.02 | 0.07 | 0.00 | 0.96 | 0.01 | 0.06 | 0.00 | 1.00 |  |  |  |  |  |  |  |  |  |  |  |  |  |  |  |  |
| Type loyalty 18 |  |  |  |  | 0.01 | 0.07 | 0.00 | 1.00 | 0.05 | 0.15 | 0.00 | 1.00 |  |  |  |  |  |  |  |  |  |  |  |  |  |  |  |  |
| Type loyalty 19 |  |  |  |  | 0.07 | 0.20 | 0.00 | 1.00 |  |  |  |  |  |  |  |  |  |  |  |  |  |  |  |  |  |  |  |  |
| Flavour loyalty 1 | 0.02 | 0.10 | 0.00 | 1.00 |  |  |  |  |  |  |  |  |  |  |  |  |  |  |  |  |  |  |  |  |  |  |  |  |
| Flavour loyalty 2 | 0.02 | 0.09 | 0.00 | 1.00 |  |  |  |  |  |  |  |  |  |  |  |  |  |  |  |  |  |  |  |  |  |  |  |  |
| Flavour loyalty 3 | 0.19 | 0.32 | 0.00 | 1.00 |  |  |  |  |  |  |  |  |  |  |  |  |  |  |  |  |  |  |  |  |  |  |  |  |
| Flavour loyalty 4 | 0.00 | 0.04 | 0.00 | 0.63 |  |  |  |  |  |  |  |  |  |  |  |  |  |  |  |  |  |  |  |  |  |  |  |  |
| Flavour loyalty 5 | 0.16 | 0.28 | 0.00 | 1.00 |  |  |  |  |  |  |  |  |  |  |  |  |  |  |  |  |  |  |  |  |  |  |  |  |
| Flavour loyalty 6 | 0.02 | 0.09 | 0.00 | 1.00 |  |  |  |  |  |  |  |  |  |  |  |  |  |  |  |  |  |  |  |  |  |  |  |  |
| Flavour loyalty 7 | 0.01 | 0.06 | 0.00 | 0.95 |  |  |  |  |  |  |  |  |  |  |  |  |  |  |  |  |  |  |  |  |  |  |  |  |
| Flavour loyalty 8 | 0.02 | 0.10 | 0.00 | 1.00 |  |  |  |  |  |  |  |  |  |  |  |  |  |  |  |  |  |  |  |  |  |  |  |  |
| Flavour loyalty 9 | 0.02 | 0.10 | 0.00 | 1.00 |  |  |  |  |  |  |  |  |  |  |  |  |  |  |  |  |  |  |  |  |  |  |  |  |
| Flavour loyalty 10 | 0.07 | 0.20 | 0.00 | 1.00 |  |  |  |  |  |  |  |  |  |  |  |  |  |  |  |  |  |  |  |  |  |  |  |  |
| Flavour loyalty 11 | 0.48 | 0.41 | 0.00 | 1.00 |  |  |  |  |  |  |  |  |  |  |  |  |  |  |  |  |  |  |  |  |  |  |  |  |

*Notes: For each household: 1. The % of store visits that result in a purchase of the good in question. 2. The number of packs bought. 3. The average daily consumption per household in centilitres in the initialisation period. 4. A [0,1] variable indicating loyalty to brand by share of volume purchased in initialisation period. 5. Same loyalty calculation for package type. 6. Same loyalty calculation for type of product.*

For multi-bags of crisps, flavoured yoghurt (large), flavoured yoghurt (multipack), natural yoghurt and everyday biscuits we were forced to make restrictions on the number of brands we could include in the model. The brand choice model (McFadden choice model) is computationally limited to approximately 80 alternatives. In table A1.2 we show how many alternatives (combinations of brand, type, flavour and size, i.e. stock keeping units (SKUs)) were eligible for analysis and how many we removed for tractability. In order to avoid selection of unavailable alternatives we removed households that had purchased these rare SKUs from our analysis, rather than just the alternatives themselves.

Table A1.2, SKU removal for analysis

| **Good** | **SKUs eligible** | **SKUs after removal** | **% of total remaining by volume** |
| --- | --- | --- | --- |
| Yoghurt (Large) | 164 | 78 | 95 |
| Yoghurt (Multipack) | 245 | 76 | 85 |
| Yoghurt (Natural) | 119 | 74 | 99 |
| Everyday biscuits | 195 | 56 | 95 |
| Crisps (Multipack) | 193 | 64 | 90 |

# Annex 2: Consumption rates under flexible consumption

Figure A2.1 Daily consumption rates for different estimates of the coefficient of consumption flexibility ($\psi$)

*Notes: This graph represents the estimate level of household consumption (y-axis) per level of inventory (x-axis). In this case household average consumption is estimated to be 8Cl / 80g per day. Products with a coefficient of consumption flexibility of 1 have a constant consumption rate of ~8Cl / 80g. For coefficients < 1 consumption is estimated to depend on inventory levels. The further the estimated coefficient from 1 the more households will consume for a given level of inventory. Here we include Ketchup and Cola as an example.*

Figure A2.2: Example daily consumption rates for different household average consumption from the initialisation period ($\psi$ = 0.128)

*Notes: Households with higher levels of average consumption in the pre-estimation period (2016) are assumed to consume greater quantities of the food or drink for a given level of inventory and estimate for the coefficient of consumption flexibility (*$\psi$*).*

# Annex 3: Estimation procedure

The estimation follows a likelihood maximisation procedure.

The likelihood function for the entire system of purchase incidence and quantity, described in the previous section, is given by:

$$L= \prod_{h} \prod_{b\in B} \prod_{t} \left( \frac{e^{U_{jt}^{h}}}{\sum e^{U_{bt}^{h}}} \right)^{D_{bt}^{h}}\left( \frac{e^{{-V}_{t}^{h}}}{1+e^{{-V}_{t}^{h}}} \right)^{1-D_{t}^{h}}\left( \frac{1}{1+e^{-V_{t}^{h}}} \right)^{D_{t}^{h}}\left( \frac{{(\lambda_{t}^{h})}^{q_{t}^{h}}}{\left( e^{\lambda_{t}^{h}}-1 \right)q_{t}^{h}!} \right)^{D_{t}^{h}} (10)$$

where:

- $D_{bt}^{h}$ = a dummy variable equal to one if *b* = *j*, the brand purchased by household *h* on trip *t*.
- $D_{t}^{h}$ = a dummy variable equal to 1 if purchase is made by household *h* on trip *t*, 0 otherwise.
- $q_{t}^{h}$ = number of units purchased by household *h* on trip *t*.

As in AN we estimate the model in two steps. First, we estimate the brand selection model for each trip by maximising the log of the first expression. We then use the coefficients from this estimation to create an ‘inclusive value’ variable for use in the purchase incidence model. We then estimate the consumption rate function (3)^[[1]](#footnote-1)^, the incidence model (7) and the quantity decision (10) together by jointly maximizing the log likelihood of the remaining three expressions in (12). The value *f* for the consumption rate function is imbedded in the likelihood function through the inventory variable. As evidence for the suitability of a flexible consumption rate function we also estimate the model with a constant consumption rate equal to the average consumption rate of the initiation period. The analysis was performed using STATA 15. For the brand choice model, we use McFadden’s choice model (1974) using the asclogit command, for the incidence we use xtlogit with household fixed effects and the quantity decision we use tpoisson with clustered standard errors and household dummies.

# Annex 4: Regression results for brand choice, purchase incidence and purchase quantity models

Table A4.1: Purchase incidence model results

|  | **Cola (all)** | **Cola (top tercile)** | **Cola (top quintile)** | **Lemonade** | **Children's cereal** | **Flavoured Yoghurt (large pots)** | **Flavoured Yoghurt (multi pack)** | **Crisps (multi pack)** | **Children's biscuits** | **Everyday Biscuits** | **Peanut Butter** | **Baked beans** | **Ketchup** | **Natural Yoghurt** |
| --- | --- | --- | --- | --- | --- | --- | --- | --- | --- | --- | --- | --- | --- | --- |
| **Incidence** |  |  |  |  |  |  |  |  |  |  |  |  |  |  |
| # obs (store visits) | 133,417 | 68,263 | 42,898 | 87,692 | 139,783 | 82,497 | 53,237 | 71,716 | 119,839 | 118,857 | 80,136 | 224,146 | 146,889 | 152,175 |
| # households | 1,638 | 811 | 501 | 1,021 | 1,720 | 1,039 | 697 | 899 | 1,471 | 1,509 | 972 | 2,722 | 1,791 | 1,882 |
| # purchases | 11,152 | 9,568 | 8,123 | 8,875 | 15,031 | 10,133 | 9,446 | 11,830 | 15,194 | 17,924 | 6,578 | 34,450 | 9,575 | 28,770 |
| % incidence per shop | 8.4% | 14.0% | 18.9% | 10.1% | 10.8% | 12.3% | 17.7% | 16.5% | 12.7% | 15.1% | 8.2% | 15.4% | 6.5% | 18.9% |
|  |  |  |  |  |  |  |  |  |  |  |  |  |  |  |
| Inventory | -0.003** | -0.003** | -0.003** | -0.003** | -0.016** | -0.017** | -0.019** | -0.068** | -0.031** | -0.027** | -0.002** | -0.007** | -0.01** | -0.019** |
|  | (0.0003) | (0.0003) | (0.00027) | (0.0004) | (0.002) | (0.00143) | (0.00165) | (0.00608) | (0.00373) | (0.00223) | (0.0002) | (0.0003) | (0.0004) | (0.001) |
| Inclusive value | 0.157** | 0.277** | 0.312** | 0.248** | 0.129** | 0.401** | 0.495** | 0.227** | 0.306** | 0.206** | 0.148** | 0.392** | 0.371** | 0.167** |
|  | (0.023) | (0.024) | (0.026) | (0.023) | (0.013) | (0.024) | (0.027) | (0.023) | (0.025) | (0.022) | (0.024) | (0.011) | (0.019) | (0.015) |
| Inc t-1 | 0.057* | 0.056* | 0.028 | -0.094** | -0.198** | 0.121** | -0.136** | -0.243** | 0.088** | -0.157** | -0.743** | -0.29** | -0.709** | -0.144** |
|  | (0.033) | (0.034) | (0.036) | (0.037) | (0.029) | (0.032) | (0.034) | (0.031) | (0.025) | (0.025) | (0.051) | (0.019) | (0.054) | (0.019) |
| Quarter 2 | 0.215** | 0.144** | 0.141** | 0.209** | -0.103** | 0.006 | -0.117** | 0.101** | -0.058** | 0.038 | -0.02 | -0.221** | 0.004 | -0.003 |
|  | (0.033) | (0.036) | (0.04) | (0.036) | (0.025) | (0.032) | (0.036) | (0.031) | (0.026) | (0.025) | (0.039) | (0.018) | (0.031) | (0.02) |
| Quarter 3 | 0.178** | 0.104** | 0.105** | 0.113** | -0.207** | -0.041 | -0.205** | -0.008 | -0.064** | 0.059** | -0.005 | -0.258** | -0.08** | -0.046** |
|  | (0.033) | (0.036) | (0.04) | (0.036) | (0.026) | (0.033) | (0.036) | (0.032) | (0.027) | (0.025) | (0.041) | (0.018) | (0.032) | (0.021) |
| Quarter 4 | 0.172** | 0.038 | 0.029 | 0.065* | -0.416** | -0.40** | -0.279** | 0.016 | -0.27** | -0.089** | -0.068 | -0.261** | -0.097** | -0.258** |
|  | (0.033) | (0.036) | (0.04) | (0.036) | (0.027) | (0.034) | (0.036) | (0.032) | (0.027) | (0.026) | (0.042) | (0.018) | (0.033) | (0.021) |

*Notes: ** is significant at the 5% level, * at the 10% level. The model is a conditional fixed effects, binary logit.*

*Table A4.1 shows the coefficients from the incidence and quantity decision regressions. The number of observations in the incidence model is the number of shopping trips taken by the consumer.*

*The effect of inventory on purchase incidence is negative across all food and drink categories: less estimated food or drink in the house leads to greater probability of purchasing that group on a given shopping trip. For all users the fact that a purchase made on the previous trip reduces the probability of incidence, with the exceptions of cola drinks, large yoghurt pots and children’s biscuits.*

*We observe seasonality in some food and drink products but not others. For all drinks purchasers there is a great probability of incidence outside q1, whereas for the food groups covered there are generally fewer purchases outside Jan-Mar.*

*The impacts of promotions on purchase incidence are observed through the “inclusive value”, which represents the expected gain from a consumer engaging in a purchase (eqn. 6). The inclusive value has a positive coefficient in all food and drink categories. A brand “menu” with different prices and promotions on offer is more likely to induce a purchase from consumers if the expected value of the purchase is larger.*

Table A4.2: Brand selection model

|  | **Cola (all)** | **Cola (top tercile)** | **Cola (top quintile)** | **Lemonade** | **Children's cereal** | **Flavoured Yoghurt (large pots)** | **Flavoured Yoghurt (multi pack)** | **Crisps (multi pack)** | **Children's biscuits** | **Everyday Biscuits** | **Peanut Butter** | **Baked beans** | **Ketchup** | **Natural Yoghurt** |
| --- | --- | --- | --- | --- | --- | --- | --- | --- | --- | --- | --- | --- | --- | --- |
| **Product selection** |  |  |  |  |  |  |  |  |  |  |  |  |  |  |
| N | 11,152 | 9,568 | 8,123 | 8,875 | 15,031 | 10,133 | 9,446 | 11,830 | 15,194 | 17,924 | 6,578 | 34,450 | 9,575 | 28,770 |
| Price | -2.28** | -2.23** | -2.35** | -1.52** | -0.92** | -0.69** | -1.07** | -1.31** | -0.86** | -0.59** | -0.20 | -2.21** | -1.81** | -0.53** |
|  | (0.239) | (0.284) | (0.343) | (0.174) | (0.138) | (0.143) | (0.101) | (0.158) | (0.091) | (0.114) | (0.15) | (0.1) | (0.171) | (0.106) |
| Total price reduction | 0.8** | 0.83** | 0.88** | 0.81** | 1.45** | 1.36** | 0.99** | 0.81** | 1.1** | 0.87** | 1.78** | 0.71** | 1.13** | 0.63** |
|  | (0.047) | (0.056) | (0.067) | (0.067) | (0.056) | (0.061) | (0.07) | (0.055) | (0.037) | (0.044) | (0.062) | (0.033) | (0.041) | (0.037) |
| Multi-Buy | 0.31** | 0.39** | 0.38** | 0.49** | 1.62** | 1.14** | 0.26** | 0.31** | 0.95** | 0.3** | 0.25 | 0.54** | 2.17** | 0.61** |
|  | (0.107) | (0.13) | (0.155) | (0.125) | (0.104) | (0.073) | (0.085) | (0.07) | (0.144) | (0.065) | (0.509) | (0.054) | (0.525) | (0.119) |
| Last alternative purchased | 2.08** | 2.07** | 2.1** | 1.76** | 2.85** | 2.52** | 2.54** | 2.41** | 2.16** | 2.36** | 2.51** | 2.2** | 1.48** | 2.84** |
|  | (0.062) | (0.071) | (0.083) | (0.074) | (0.081) | (0.076) | (0.099) | (0.071) | (0.047) | (0.049) | (0.078) | (0.042) | (0.055) | (0.052) |
| Brand loyalty | 1.91** | 2.07** | 2.17** | 2.57** | 2.19** | 1.37** | 2.07** | 1.63** | 2.35** | 1.33** | 2.85** | 2.64** | 2.38** | 2.04** |
|  | (0.074) | (0.1) | (0.124) | (0.085) | (0.09) | (0.083) | (0.127) | (0.089) | (0.074) | (0.068) | (0.188) | (0.064) | (0.12) | (0.068) |
| Size loyalty | 1.36** | 1.49** | 1.55** | 1.79** |  |  |  |  | 1.63** | 0.21** | 1.22** | 1.39** | 1.09** | 1.48** |
|  | (0.081) | (0.112) | (0.142) | (0.202) |  |  |  |  | (0.455) | (0.057) | (0.097) | (0.059) | (0.184) | (0.199) |
| Type loyalty |  |  |  |  | 0.59** | 0.83** | 0.86** | 1.68** | 0.5** | 2.48** | 2** | 2.12** | 1.91** | 1.55** |
|  |  |  |  |  | (0.228) | (0.087) | (0.113) | (0.135) | (0.12) | (0.068) | (0.097) | (0.072) | (0.203) | (0.052) |
| Flavour loyalty |  |  |  |  |  | 1.943** | 1.826** | 2.403** |  |  |  |  |  |  |
|  |  |  |  |  |  | (0.087) | (0.094) | (0.101) |  |  |  |  |  |  |

*Notes: ** significant at the 5% level, * at the 10% level. This regression is an alternative-specific conditional logit 9mcFadden’s choice) model. In addition to the alternative specific variables such as price and promotion presented here, one can also include variables that only vary between cases. In our regression we include household level average consumption which interact with the choice for each brand. The estimates for these coefficients are available on request.*

*For all food categories a total price reduction offer is more likely to result in a purchase of a particular item: if at least one brand is on promotion the expected value of making a purchase will increase. For all product categories the presence of a TPR promotion on a brand increases demand for that brand. This effect is frequently stronger for TPRs than multi-buy purchases, only ketchup and children’s cereal have multi-buy coefficients larger than TPRs.*

*Brand loyalty is found to be important in a consumer’s decision, such as Heinz for baked beans and ketchup, Pepsi for cola. In baked bean, ketchup and cereal estimations the “type” is also important. The type for cereal is, for example, chocolate rice shaped rather than honey hoops or chocolate flakes, implying consumers are more likely to switch brand from, say, Kellogg’s to own brand than choose a different type of chocolate cereal. In yoghurts and crisps, flavour loyalty is also a statistically significant factor. Households show loyalty on the basis of flavour and brand.*

*The Last alternative purchased variable controls for consumers’ persistence in shopping habits. If they bought that exact item (brand, size, flavour and type) last time how likely is it they buy it again? The alternative purchased consistently significant result reflects consistency in consumer behaviour beyond the loyalty for brands, types and sizes: consumers will frequently purchase exactly the same product – brand, container shape, size, flavourings – as they did for the previous purchase.*

*In order to assess the estimated impact of price promotions on purchase incidence we must calculate the increase in the inclusive value that price promotions create. There are two influencing aspects to a PP: average price change and the promotion itself (bright colours and multi-buy deals).*

Table A4.3: Purchase quantity model

|  |  | **Cola (all consumers)** | **Cola (top tercile)** | **Cola (top quintile)** | **Lemonade** | **Children's cereal** | **Flavoured Yoghurt (large pots)** | **Flavoured Yoghurt (multi pack)** | **Crisps (multi pack)** | **Children's biscuits** | **Everyday Biscuits** | **Peanut Butter** | **Baked beans** | **Ketchup** | **Natural Yoghurt** |
| --- | --- | --- | --- | --- | --- | --- | --- | --- | --- | --- | --- | --- | --- | --- | --- |
| **Quantity decision** |  |  |  |  |  |  |  |  |  |  |  |  |  |  |  |
| N obs |  | 11,152 | 9,568 | 8,123 | 8,875 | 15,031 | 10,133 | 9,446 | 11,830 | 15,194 | 17,924 | 6,578 | 34,450 | 9,575 | 28,770 |
|  |  |  |  |  |  |  |  |  |  |  |  |  |  |  |  |
| Log price |  | -0.211* | -0.194 | -0.2 | -0.246** | -0.556** | -0.305** | -0.239** | -0.225** | -0.198** | -0.336** | -0.768** | -0.33** | -0.249 | -0.168** |
|  |  | (0.114) | (0.119) | (0.128) | (0.087) | (0.097) | (0.115) | (0.077) | (0.105) | (0.067) | (0.051) | (0.213) | (0.052) | (0.438) | (0.067) |
| Total price reduction |  | 0.091* | 0.105** | 0.115** | 0.079* | 0.522** | 0.299** | 0.337** | 0.361** | 0.277** | 0.316** | 0.672** | 0.269** | 0.736** | 0.23** |
|  |  | (0.051) | (0.05) | (0.051) | (0.045) | (0.073) | (0.064) | (0.056) | (0.065) | (0.034) | (0.035) | (0.132) | (0.023) | (0.144) | (0.039) |
| Multi-Buy |  | 0.33** | 0.317** | 0.291** | 0.489** | 1.393** | 0.914** | 0.822** | 1.064** | 0.844** | 0.675** | 2.675** | 0.745** | 2.534** | 0.815** |
|  |  | (0.066) | (0.065) | (0.064) | (0.084) | (0.098) | (0.084) | (0.084) | (0.081) | (0.093) | (0.043) | (0.209) | (0.037) | (0.514) | (0.086) |
| Inventory |  | -0.0004** | -0.0004** | -0.0004** | -0.00004 | -0.0075** | -0.0016** | -0.0037** | -0.004** | -0.0184** | -0.0073** | -0.0001** | -0.0002** | -0.0034** | -0.0034** |
|  |  | (0.0002) | (0.0002) | (0.0002) | (0.0004) | (0.006) | (0.0004) | (0.0018) | (0.0012) | (0.009) | (0.0027) | (0.0001) | (0.0001) | (0.001) | (0.0018) |
| Size |  | -0.003** | -0.003** | -0.002** | -0.019 | -0.016** | -0.017** | -0.014** | -0.042** | -0.029** | -0.023** | -0.024** | -0.019** | -0.016** | -0.024** |
|  |  | (0.0004) | (0.0004) | (0.0004) | (0.088) | (0.002) | (0.004) | (0.002) | (0.006) | (0.007) | (0.002) | (0.006) | (0.0006) | (0.006) | (0.002) |
| Household level means | Average quantity purchased | 0.366** | 0.355** | 0.344** | 0.562** | 0.863** | 0.602** | 0.542** | 0.739** | 0.599** | 0.371** | 1.697** | 0.358** | 1.408** | 0.56** |
|  |  | (0.05) | (0.05) | (0.04) | (0.05) | (0.12) | (0.06) | (0.07) | (0.08) | (0.07) | (0.03) | (0.08) | (0.027) | (0.11) | (0.06) |
|  | Log price | -0.049 | -0.074 | -0.065 | 0.265* | 0.465** | 0.24 | 0.235* | -0.003 | 0.106 | -0.023 | 0.533** | 0.039 | -0.487 | -0.052 |
|  |  | (0.16) | (0.16) | (0.17) | (0.15) | (0.15) | (0.18) | (0.13) | (0.13) | (0.09) | (0.08) | (0.24) | (0.06) | (0.54) | (0.11) |
|  | Total price reduction | 0.145 | 0.194 | 0.214 | -0.003 | 0.098 | 0.024 | -0.205* | 0.099 | -0.149** | 0.146* | -0.145 | -0.202** | -0.582** | -0.012 |
|  |  | (0.16) | (0.17) | (0.18) | (0.15) | (0.14) | (0.1) | (0.12) | (0.09) | (0.06) | (0.08) | (0.28) | (0.06) | (0.27) | (0.15) |
|  | Multi-Buy | 0.200 | 0.155 | 0.154 | 0.203 | -0.652** | -0.236** | -0.058 | -0.457** | -0.205 | -0.21** | -2.923** | -0.183* | -3.918 | -0.091 |
|  |  | (0.16) | (0.17) | (0.18) | (0.2) | (0.21) | (0.1) | (0.17) | (0.12) | (0.22) | (0.07) | (0.9) | (0.11) | (3.44) | (0.18) |
|  | Inventory | -0.01** | -0.009** | -0.009** | 0.0004 | -0.031** | -0.0103** | -0.0169** | -0.1022** | -0.0761** | -0.0191** | 0.0117** | 0.0003 | 0.004 | -0.0311** |
|  |  | (0.0004) | (0.00039) | (0.00043) | (0.1165) | (0.003) | (0.009) | (0.0031) | (0.0035) | (0.0118) | (0.0018) | (0.0056) | (0.0005) | (0.0073) | (0.0028) |
|  | Size | 0.0003 | 0.00019 | -0.0001 | 0.078 | 0.004 | 0.045** | 0.009** | 0.023** | 0.013 | 0.008** | 0.02** | 0.008** | 0.005 | 0.014** |
|  |  | (0.7) | (0.75) | (0.77) | (20.17) | (0.41) | (0.6) | (0.46) | (0.3) | (0.25) | (0.28) | (0.66) | (0.17) | (1.01) | (0.38) |
|  | Constant | -2.167** | -2.105** | -2.027** | -13.021 | -2.18** | -3.014** | -1.062** | -2.294** | -1.31** | -1.844** | -4.237** | -1.496** | -6.083** | -2.01** |
|  |  | (0.7) | (0.75) | (0.77) | (20.17) | (0.41) | (0.6) | (0.46) | (0.3) | (0.25) | (0.28) | (0.66) | (0.17) | (1.01) | (0.38) |

*** is significant at the 5% level, * at the 10% level. These results are from a Poisson regression model that is truncated at 0. In order to replicate the fixed effects regression, we use the Mundlack procedure. The multi buy promotions are statistically significant in increasing the probability that a consumer will buy a higher quantity of their chosen product. This is naturally determined by the promotion, which mandates the consumer to purchase more than one item to get hold of the deal. For TPRs, where there is no mandate on quantity, consumers also increase their quantity purchases. For all products the marginal effect is higher for multi-buy promotions than for TPRs.*

*The model fit from including flexible consumption in the quantity decision, shown in Table 2, improves for all products except for lemonade, multipack flavoured yoghurt and baked beans. So, conditional on a purchase being made and the brand chosen, the quantity purchased model fit is not improved by allowing consumption to be flexible for these products. We can see this in the statistical significance of the inventory coefficient: the quantity decision is estimated to depend on household inventory with a negative coefficient with all products but lemonade and baked beans. However, for both products the incidence decision shows a larger increase in log-likelihood so the overall model fit is improved with flexible consumption.*

*The “Pack size chosen” has a negative effect on the quantity purchased, because if someone is buying a 12-pack of canned cola, they need to buy fewer to get to their preferred inventory than if they buy a 6-pack.*

1. *f* from equation (3) is estimated manually by selecting the value of *f* that generates the highest overall likelihood for the model. [↑](#footnote-ref-1)
